# Noncoding RNAs in Pediatric Solid Tumors: Advances in Understanding and Critical Knowledge Gaps

**DOI:** 10.3390/cells15050465

**Published:** 2026-03-05

**Authors:** Graham Duff, Christine Mella, Alexa Amato-Loudon, Meredith Farrell, Rachael Aldridge, Hope C. Ball

**Affiliations:** 1 Department of Biology, The University of Akron, 302 Buchtel Common, Akron, OH 44325, USA; grahamduff01@gmail.com; 2 Division of Hematology Oncology, Akron Children’s Hospital, One Perkins Square, Akron, OH 44308, USA; cmella@akronchildrens.org (C.M.); aa324317@ohio.edu (A.A.-L.); mferrel@neomed.edu (M.F.); rachael.aldridge@live.mercer.edu (R.A.); 3 Heritage College of Osteopathic Medicine, Ohio University, 191 West Union Street, Athens, OH 45701, USA; 4 College of Medicine, Northeast Ohio Medical University, 4029 State Route 44, Rootstown, OH 44272, USA; 5 College of Medicine, Mercer University School of Medicine, 1550 College St., Macon, GA 31207, USA; 6 Rebecca D. Considine Research Institute, Akron Children’s Hospital, One Perkins Square, Akron, OH 44308, USA

**Keywords:** non-coding RNA, epigenetics, pediatrics, cancer, solid tumors, drug resistance

## Abstract

The etiology of pediatric cancers is unique, stemming from developmental dysregulation rather than acquired mutations from carcinogenic exposure. These diseases demonstrate vastly different underlying genetic and epigenetic alterations and unique tissue microenvironments which are only now beginning to be explored. While many pediatric cancers have seen improved overall and event-free survival rates thanks to innovations in diagnosis and treatment, many have seen little to no improvement in patient outcomes. This highlights a critical need for additional research into the underlying genetic and epigenetic alterations in these pathologies. Non-coding RNAs (ncRNAs) are functional RNA molecules known to regulate gene expression at epigenetic, transcriptional, and translational levels and can serve as biomarkers of disease. Here, we examine current knowledge of the roles of microRNAs (miRNAs), long non-coding RNAs (lncRNAs), and circular RNAs (circRNAs) in the onset, progression, and therapeutic response of pediatric solid tumors. We discuss the current and future potential and pitfalls of these molecules as therapeutics and biomarkers and highlight critical knowledge gaps where future research might provide insight to improve current therapeutic strategies and improve clinical outcomes.

## 1. Introduction

Cancer affects both adult and pediatric patient populations and is a major global socioeconomic health problem [1,2]. Adult cancers often arise from accumulated genetic and/or epigenetic mutations affecting epithelial cell transcription/translation, cycle regulation, or DNA damage repair mechanisms [3,4]. The etiology of pediatric cancers, however, lies in the developmental dysregulation of mesoderm-, ectoderm-, or endoderm-originating cells, resulting in fusion genes or oncogenic changes in stem cell populations or the tissue microenvironment (TME) [5,6,7]. Furthermore, the pediatric cancer-associated gene mutations are significantly different from adult-onset counterparts, and the mechanisms of disease onset, metastasis, and therapeutic response remain poorly understood.

In the pediatric population, cancer stands as the second leading cause of death, second only to injury-related mortalities [8,9]. Solid tumors comprise around 60% of all diagnosed pediatric cancers and include intracranial (central nervous system CNS, neuroblastoma) and extracranial (Wilms, hepatoblastoma, retinoblastoma, soft tissue sarcomas, rhabdomyosarcomas, osteosarcoma, Ewings sarcoma) tumors [10,11]. Improvements in chemotherapy/radiotherapy regimens, surgical approaches, and targeted gene therapies have improved childhood cancer survival overall, but treatment options remain limited for children with metastatic, relapsed, or therapy-resistant disease [12,13]. Recent years have seen a significant rise in the use of next-generation sequencing (NGS) assessment of patient samples, leading to improved understanding of the underlying mutations and pathways in many pediatric cancer types. In cancers that lack common risk factors, demonstrate high genetic heterogeneity, or arise spontaneously, studies have focused on elucidating alternative mechanisms of developmental genetic/epigenetic regulation such as non-coding RNAs (ncRNAs).

ncRNAs have known roles in cancer pathogenesis where they regulate oncogenic pathways, serve as potential therapeutic targets, and as biomarkers for disease sub-type and/or stage [14,15,16]. Here, we provide an updated review on the role of ncRNAs in the pediatric solid tumors medulloblastoma, retinoblastoma, rhabdomyosarcoma, hepatoblastoma, osteosarcoma, and Ewing sarcoma. These malignancies were selected based on diagnostic prevalence and scientific advancement in the roles on ncRNAs for clinical and therapeutic applications. We provide in-depth insight into the roles of miRNAs, lncRNAs, and circRNAs in tumor biogenesis, progression, metastasis, and therapy resistance, and identify areas of interest that could benefit from additional scientific study.

## 2. Materials and Methods

A PUBMED literature review was completed to search for original research articles and recent in-depth reviews detailing the role of various non-coding RNAs on solid tumor onset, progression, metastasis, clinical correlation, and therapeutics in the pediatric population. Database searches utilized the specific disease name, pediatric, and the key words “pathogenesis,” “prevalence,” “therapeutic treatment,” “outcome,” “survival,” “miRNA,” “long non-coding RNA,” circRNA,” “non-coding RNA,” “non-coding RNA and clinical correlate,” and “RNA pediatric cancer biomarker.” Figures were created using BioRender.com (Toronto, ON, Canada)

## 3. Biogenesis of miRNAs, lncRNAs, and circRNAs and Their Roles in Health and Disease

ncRNAs are RNA molecules that do not have protein-coding functions, but regulate gene expression at epigenetic, transcriptional, and translational levels [17]. There are many subtypes of ncRNAs classified according to size and three-dimensional structure. These include microRNAs (miRNAs), small nucleolar RNAs (snoRNAs), circular RNAs (circRNAs), Piwi-interacting RNAs (piRNAs), small interfering RNAs (siRNAs), small cytoplasmic RNAs (scRNAs), and long non-coding RNAs (lncRNAs) [18]. All ncRNA subtypes demonstrate critical roles in cell and tissue homeostasis, inter- and intra-cellular signaling, gene expression, and pathway activation, and their dysregulation contributes to the pathogenesis of human disease [19].

**Figure 1:**
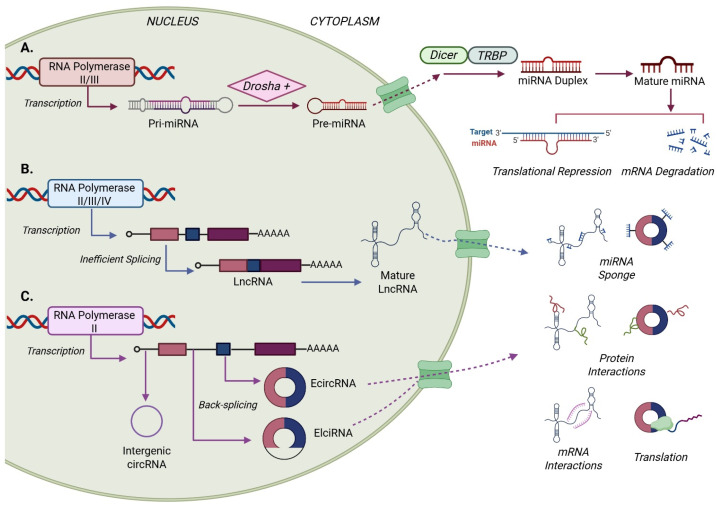
Biogenesis and functions of miRNAs, lncRNAs, and circRNAs. (**A**). miRNAs are transcribed by RNA polymerase II/III and further processed by the Drosha complex prior to nuclear export. In the cytoplasm, pre-miRNAs are processed to mature miRNAs by the enzymes Dicer and Transactivation response RNA binding protein (TRBP). Mature miRNAs become part of RNA-silencing complexes (RISCs) to mediate translational repression and degradation of mRNA. (**B**). lncRNAs are the result of inefficient splicing. In the nucleus, they obtain a 5′ cap and 3′ poly-A tail and form their unique three-dimensional structures. After cytoplasmic transport, lncRNAs interact with proteins, mRNAs, and miRNAs to regulate gene and pathway function. (**C**). circRNAs are formed from back-spliced mRNA templates forming intergenic, EcircRNA, and ElciRNA (among others). Once transported to the cytoplasm, circRNAs modulate gene and pathway function via sponging interactions with various proteins and/or miRNAs or are translated.

**Figure 2:**
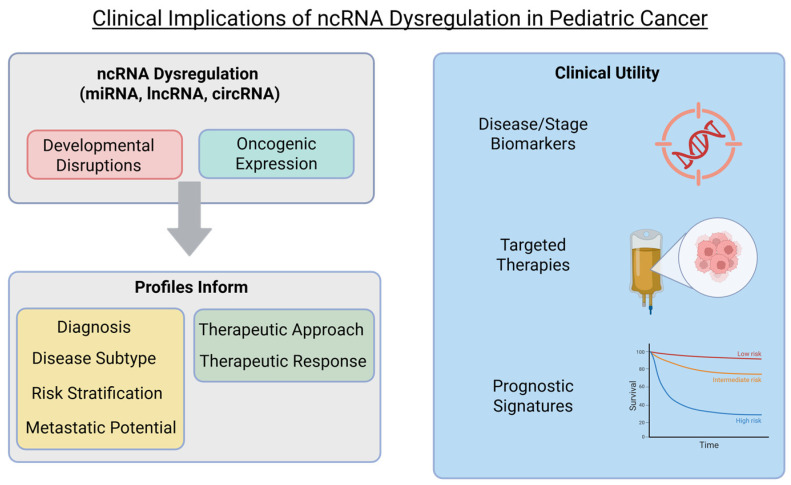
ncRNAs inform diagnosis, treatment, and prognosis of pediatric solid tumors. Next generation sequencing of pediatric cancer lines and patient samples continue to improve knowledge surrounding ncRNA profiles and alterations with disease type, stage, and therapeutic response. These in turn have the potential to inform discoveries of disease-specific biomarkers and novel therapeutic targets.

Micro-RNAs (miRNAs) are small ncRNAs measuring approximately 22 nucleotides (nt) in length that are capable of promoting mRNA degradation via the formation of RNA-induced silencing complex (RISC) or causing translational repression through imperfect binding to the 3′ untranslated regions (UTR) of mRNA. miRNA biogenesis begins with RNA polymerase II/III transcription of pri-miRNAs which are cleaved to form pre-miRNAs before being exported to the cytoplasm for Dicer-associated processing (Figure 1) [20]. Once within a RISC, miRNAs can bind mRNA targets to induce endonuclease degradation or 3′UTRs for translational repression or interact with other ncRNAs to regulate expression profiles and/or signaling pathways [21]. Their role in health and disease is influenced by the tight regulation of expression in cell-type, developmental, spatial, and temporal manners [22]. In pediatric cancer, miRNAs are of increasing interest due to their roles as regulators of oncogenic pathways, apoptosis, and cell metabolism, as well as their potential as biomarkers and precision therapeutic targets [23,24]. Next generation sequencing studies of cell lines and patient tumors are continuing to identify novel miRNAs and better elucidate how alterations in miRNA expression correlate with clinical diagnosis, therapeutic response, and overall survival (Figure 2) [25].

Long non-coding RNAs (lncRNAs) are modular RNAs greater than 200 nt in length that often comprise repeating units and demonstrate tissue- and cell lineage-specific spatiotemporal expression patterns. lncRNAs are transcribed by RNA polymerases I/II/III and undergo inefficient splicing to form mature lncRNAs (Figure 1) [26,27,28]. Mature lncRNAs influence normal cell differentiation, development, and play a role in the pathology of disease [29]. In cancer, lncRNAs modulate cell metabolism, intra- and intercellular signaling, transcription, and oncogenic or tumor-suppressive processes through genetic/epigenetic mechanisms such as miRNA sponging, RNA-RNA, RNA-protein, and/or RNA-DNA interactions or via chromatin or histone associations (Figure 2) [30,31].

Circular RNAs (circRNAs) are formed from single-stranded, covalently bonded circular RNA created from alternative splicing of PolII-transcribed pre-mRNAs (Figure 1) [32]. The sequence types that are included during splicing lead to the generation of three distinct circRNA sub-types: EIciRNA (containing exon–intron sequences), ciRNAs (containing intron-only sequences), or ecircRNAs (containing exon sequences only) [33]. circRNAs function as miRNA sponges to modulate gene transcription, or interact with RNA-binding proteins, proteases, and/or transcription factors to regulate gene/protein function and stability [34,35]. circRNAs play a role in normal cell differentiation, embryonic development, and organogenesis and have been implicated in the onset and progression of diseases including Alzheimer’s, Parkinson’s, diabetes, and cancer, where they contribute to tumorigenesis by enhancing cell proliferation and invasiveness and are being examined as potential cancer biomarkers and as potential therapeutic targets (Figure 2) [36,37].

## 4. ncRNAs in Pediatric Solid Tumors

### 4.1. Medulloblastoma

Medulloblastoma (MB) is the most common undifferentiated central nervous system (CNS) embryonal neuroectodermal tumor that represents between 15 and 20% of all childhood CNS tumors [38,39]. Commonly arising in the cerebellum, these cancers are aggressive with metastatic lesions disseminating via cerebral spinal fluid (CSF) to the spine and leptomeningeal and subarachnoid spaces [40,41]. MB is divided into four sub-groups based on histological and molecular characteristics: Sonic-Hedgehog-amplified (SHH), Wingless amplified (WNT), and non-WNT/non-SHH groups 3 and 4 [42,43]. Of these groups, SHH-amplified and non-WNT/non-SHH are the most common. Common symptoms at presentation include persistent morning headaches, nausea, ataxia, and unexplained changes in coordination and/or behavior [44,45]. Standard-of-care therapy is interdisciplinary and includes adjuvant/neo-adjuvant chemotherapy, gross surgical resection, and craniospinal irradiation [46,47]. Overall survival rates and risk of recurrence vary greatly between subgroups. Diagnoses of WNT- and SHH-amplified MB are considered more favorable than non-WNT/non-SHH amplified group 3 and group 4, which demonstrate the highest rate of metastasis at diagnosis (40–45%), the highest risk of recurrence, and worst survival rates of all subtypes [48,49,50]. Recent research has greatly advanced our understanding of the biological and pathological roles of ncRNAs in MB (Table 1).

Of all MB-associated ncRNAs, miRNAs are the best known, and their roles in apoptosis, tumor suppression, metastasis, and associations with specific sub-groups are beginning to be elucidated. The four sub-groups of MB are known to demonstrate unique miRNA profiles that correlate to prognosis and clinical outcome. Within the WNT-amplified subgroup, miRNAs-206, -183, -133b, -128a/b, -148a, and -383 are significantly reduced, resulting in overexpression of their oncogenic targets and increased cell survival, proliferation, and migration [51,52,53,54,55]. In SHH amplified MB, let-7 has been identified as a potential biomarker, and miRNA-466-3p has been shown to be downregulated, leading to enhanced epithelial–mesenchymal cell transition via unchecked modulation of target genes neuropilin 2 (*Nrp2*) and vascular endothelial growth factor alpha (*Vegfa*) [56,57]. Also, inhibition of miRNAs-18a, -19a, -20a, -21, -25, and -106 (together, the miRNA-17-92 cluster) has been shown to significantly inhibit cancer proliferation and tumor progression [58]. Group 3 and 4 non-WNT/non-SHH amplified are the most aggressive MB sub-groups and share alterations in chromosome 17 structure and/or number [59]. In Group 3, elevated expression of the miRNA cluster 183-96-182 promotes cell migration and alters cell cycle regulation in both mouse MB models and in human cell lines and tissues [60,61]. Furthermore, studies demonstrate downregulation of the miRNA-30 family in Group 3 MB results in reduced cancer cell autophagy, which can be restored with lentiviral miRNA-30a treatment [62,63]. Group 4 MB cases demonstrate loss of miRNA-4521, leading to increased cell proliferation via overexpression of oncogenic transcription factor forkhead box M1 (*FOXM1*) [64,65]. Also, Group 4 MBs commonly overexpress miRNA-187-3p and miRNA-660-5p, which have been linked to poor survival rates [66].

Long non-coding RNAs (lncRNAs) are another MB-associated ncRNA with oncogenic and tumor suppressive functions [67,68]. lncRNAs HOTAIR (HOX transcript antisense RNA) and TP73-AS1 (P73 antisense RNA 1T) have both been found to be significantly enhanced in MB tumors and cell lines, where they function as miRNA sponges to promote in vivo tumorgenicity and in vitro cancer cell migration, viability, and proliferation [69,70,71]. Several other lncRNAs have been identified as drivers of MB progression and metastasis. These include SPRY4-IT1 (SPRIGHTLY), LOXL1-AS1 (LOXL1 antisense RNA1), Linc-NeD125 (neuronal differentiation long intergenic non-coding RNA NeD125), CCAT1 (colon cancer associated transcript 1), CRNDE (colorectal neoplasia differentially expressed), HHIP-AS1 (Hedgehog interacting protein antisense 1), and UCA1 (Urothelial carcinoma associated 1) [72,73,74,75,76,77,78,79,80]. Other lncRNAs have been identified with links to specific MB sub-groups. For instance, lncRNAs lncMB1, lncMB2, and lncMB3 have been linked to the aggressive Group 3 MYC proto-oncogen (MYC) subgroup, and LOXL1-AS1 is associated with poor prognosis in SHH MB. Finally, lncRNA NEAT1 (nuclear enriched abundant transcript 1) is associated with MB chemo- and apoptotic resistance, and Nkx2-2as (Nkx2-2 antisense RNA1) has been shown to suppress MB cell proliferation and invasion in vitro [81,82].

While circRNAs are less well-known in the pathophysiology of MB, several have been identified and associated with the disease. One of the most studied is Circ_SKA3, which is upregulated in MB tissues compared to normal cerebellum, and has been shown to promote tumor invasion by maintaining cyclin-dependent kinase 6 activity [83,84]. Other upregulated circRNAs include circ_DTL, circ_KDHRBS2, circ-103128, and circ_63706, which have been associated with altered lipid metabolism in SHH subgroup MB [85,86,87]. Other circRNAs such as circ_CRTAM, circ_MAP3K5, circ_RIMS1-1, and circ_FLT3-1 are significantly downregulated in MB, but their roles and mechanisms remain largely unknown [88].

**Table 1:** Experimentally validated ncRNAs that contribute to MB progression and therapeutic resistance.

Disease/ Subgroup	Expression	Non-Coding RNA	Validated Target Gene(s)	Functional Role	Reference(s)
MB (WNT)	Downregulated	miRNAs-206, -183, -133b, -128a/b, -148a, -383	N/A (Oncogenic targets)	Increases survival, proliferation, and migration	[51,52,53,54,55]
MB (SHH)	Downregulated	miRNA-466-3p	NRP2 and VEGFA	Enhances EMT via unchecked modulation	[57]
MB (SHH)	Upregulated	miRNA-17-92 cluster (18a, 19a, 20a, 21, 25, 106)	N/A	Promotes proliferation and tumor progression	[58]
MB (Group 3)	Upregulated	miRNA cluster 183-96-182	Cell cycle regulation	Promotes migration; alters cell cycle	[60,61]
MB (Group 3)	Downregulated	miRNA-30 family	Autophagy (process)	Reduces cancer cell autophagy	[62,63]
MB (Group 4)	Downregulated	miRNA-4521	FOXM1	Increases proliferation	[64,65]
MB	Upregulated	lncRNA HOTAIR/TP73-AS1	miRNA Sponges	Promotes tumorigenicity, migration, and viability	[69,70,71]
MB (Group 3)	Upregulated	lncMB1, lncMB2, lncMB3	MYC	Associated with aggressive MYC subgroup	[81]
MB (SHH)	Upregulated	lncRNA LOXL1-AS1	N/A	Linked to poor prognosis	[74,81]
MB	Upregulated	lncRNA NEAT1	N/A	Linked to chemo- and apoptotic resistance	[81]
MB	Downregulated	lncRNA Nkx2-2as	N/A	Suppresses proliferation and invasion	[82]
MB	Upregulated	circ_SKA3	CDK6	Promotes tumor invasion	[83,84]
MB (SHH)	Upregulated	circ_DTL, _KDHRBS2, -103128, _63706	Lipid metabolism	Alters metabolic regulation	[85,86,87]

### 4.2. Retinoblastoma

Retinoblastoma (RB) is the most common type of ocular malignancy affecting children between two and four years of age [89]. While RB does not demonstrate gender predisposition, incidence does show geographical and socioeconomic correlations. Geographically, higher incidence rates occur in the Andean regions of Latin America, Asia, Western Europe, and in the eastern regions of sub-Saharan Africa [90,91]. Socioeconomically, incidences rates and age at diagnosis are both higher in lower-income nations [92]. Global prevalence is on the rise with a 34% increase in diagnoses since 1990 [93]. RB is caused by de novo inactivation of the retinoblastoma 1 gene (*RB1*) on chromosome 13q14, resulting in dysregulated DNA replication, cell differentiation, and cell senescence, and impairments in chromatin remodeling [94,95]. Inactivation can occur through *RB1* mutations or exon deletion and can be inherited (autosomal dominant, ~40% of cases) or somatic (~60% of cases) in nature [96,97]. Mode of inheritance influences clinical presentation, with bilateral disease associated with inherited mutations and unilateral with somatic [98]. Along with *RB1*, activating mutations in MYCN proto-oncogene (*MYCN*) are also associated with RB onset and progression [94]. Common clinical presentation of primary RB is an abnormal whitish retinal reflection (leukocoria), but other symptoms may include bilateral/unilateral strabismus or vision changes [99,100]. If metastasized at time of diagnosis, alternative presentations may include orbital swelling, intraocular pressure changes or hemorrhage, lymph node enlargement, or bone pain [101,102]. Treatment strategies vary widely based on accessibility of care and include enucleation as well as intravitreal, intracameral, periocular, or intra-arterial delivery of chemotherapy agents such as Melphalan, Topotecan and/or Carboplatin [103,104]. Additional systemic chemotherapy can also be applied, commonly employing Vincristine, Etoposide, and/or Carboplatin agents [105,106]. Overall survival rates for patients with primary RB are ~90% for heritable RB1 mutations and ~95% for RB of somatic origin [96]. The most common causes of RB-related mortality are metastatic disease or recurrent primary RB [107,108]. Medical innovations to uncover innovative treatment options continue to be explored, including at the role of ncRNAs and their respective mechanisms of action (Table 2).

Expression profiles and pathway analyses have yielded tremendous information regarding the pro- and anti-tumor roles of miRNAs in RB proliferation, therapeutic resistance, and invasion. For instance, miRNA-320a has been found to promote RB cell proliferation and apoptotic resistance through the inhibition of tumor suppressor candidate 3 (*TUSC3*) [109]. miRNA-889-3p also promotes RB tumor growth and inhibits apoptosis through activation of the c-Jun N-terminal kinase (*JNK*)/mitogen-activated protein kinase (*MAPK*)/extracellular signal-related kinase (*ERK*) pathway [110]. Studies have shown miRNA-25-3p directly targets PTEN, enhancing epithelial-to-mesenchymal transition (EMT) and increasing cell migration [111]. miRNA-141-3p is also upregulated in RB, and promotes angiogenesis and cell proliferation by inhibiting sushi domain-containing protein 2 (*SUSD2*) [112]. miRNA-222 is also upregulated and promotes chemotherapy resistance via the inhibition of tumor suppressor Von Hippel–Lindau (*VHL*) [113]. Similarly, miRNAs-224-3p and-492 are upregulated and function to increase cancer invasion by suppressing the functions of enzyme large tumor suppressor kinase 2 (*LATS2*) [114,115]. Finally, RB exosome-derived miRNA-92a-3p enhances tumor-associated angiogenesis through suppression of Krüppel-like factor 2 (*KLF2*) [116]. Conversely, tumor suppressor miRNAs-34a and -34b-5p both inhibit neurogenic locus notch homology protein (*NOTCH*) signaling, which increases chemosensitivity and suppresses RB invasion, respectively [117,118]. Tumor suppressor miRNA-153-3p is downregulated in RB, resulting in unchecked expression of the insulin-like growth factor 1 receptor (*IGFR1*)/rapidly accelerated fibrosarcoma (*Raf*)/mitogen-activated protein kinase kinase 1 (*MEK*) and phosphoinositide 3-kinase (*PI3K*)/AKT serine/threonine kinase 1 (*AKT*) pathways [119]. Similarly, suppression of miRNA-361-3p results in the increased expression of Sonic Hedgehog transcription factors GLI family zinc fingers 1 and 3 (*GLI1/GLI3*) [120].

Studies into the tumorigenic or tumor-suppressive functions of lncRNAs in RB pathogenesis have greatly expanded in the past few years. For example, lncRNAs-AFAP1-AS1 and -BANCR are elevated in RB and correlated with increased optic nerve invasion and tumor size [121,122]. lncRNA-MALAT1 upregulates STAT3 expression via sponging inhibition of miRNA-20b-5p and also contributes to chemotherapy resistance by sponging miRNAs-124 and -598-3p [123,124]. lncRNA-NEAT1 also promotes RB tumor growth by sponging tumor suppressive miRNAs miRNA-124, -3619-5p, 24-3p, -106a, and -148-3p [125,126]. The lncRNA-XIST contributes to RB EMT via repression of miRNA-101 and increased proliferation and invasion through the inhibition of miRNAs-140-5p and -191-5p [127,128]. In RB, the expression of lncRNAs-TMPO-AS1 and -MIR17HG is increased in the hypoxic TME, where they promote invasion and disrupt cell cycle regulation, respectively [129,130]. Similarly, lncRNA-ANRIL is upregulated by *HIF1α* and contributes to chemotherapeutic resistance by sponging miRNA-328 [131]. Other significantly upregulated lncRNAs associated with RB include lncRNA-SNHG20, -HOTAIR, -PVT1, -LINC00202, and MIMT1 [132,133,134,135,136]. Tumor suppressor lncRNAs have also been identified in RB cell lines and tissues. lncRNAs-MT1JP, -MBLN1, and -MEG3 all target various aspects of the WNT/β-catenin pathway [137,138,139]. Two additional lncRNAs demonstrate clinical correlations in RB: lncRNAs -BDNF-AS and -NKILA are correlated with poor overall survival and tumor grade/size, respectively [140,141].

Recent studies have added to the body of literature regarding the role of circRNAs in RB onset, progression, and therapeutic response. Circ-DHDDS (has_circ_0000034) promotes RB progression via sponging tumor suppressor miRNA-361-3p [142,143]. circ-FAM158A (has_circ_0000527) promotes RB onset, metastasis, and optic nerve invasion through miRNA sponging, which increases XIAP and smad family member 2 (SMAD2) expression and regulates the expression of low-density lipoprotein receptor-related protein 6 (*LRP6*), [144,145]. Oncogenic circ-E2F3 (has-circ_0075804) sponges miRNA-204-5p to regulate rho-associated protein kinase 1 (*ROCK1*), and enhances RB motility and invasion via the regulation of LIM and SH3 protein 1 (*LASP1*) expression [146,147]. circ_RNF20 (has_circ_0087784) is also upregulated and promotes RB invasion by sponging miRNA-132-3p and increases *PAX6* expression [148]. Tumor suppressor circRNAs have also been identified in RB. lncRNA circ-TET1 (has_circ_0093996) targets the WNT/β-catenin pathway by inhibiting miRNAs-492 and -484-3p [149]. Similarly, circ-SHPRH (has_circ_0001649) promotes cell apoptosis by promoting phosphorylyation and activation of AKT and mTOR [150]. circ-MKLN1 (has_circ_0082415) curbs RB-associated cell invasion by sponging tumorigenic miRNA-425-5p, and circ-CUL2 (hsa_circ_0000234) inhibits the expression of E2F transcription factor 2 (*E2F2*) to reduce RB-associated cellular proliferation and migration [151,152].

**Table 2:** Experimentally validated ncRNAs that contribute to RB progression and invasion.

Disease/ Subgroup	Expression	Non-Coding RNA	Validated Target Gene(s)	Functional Role	Reference(s)
RB	Upregulated	miRNA-320a	TUSC3	Promotes proliferation and apoptotic resistance	[109]
RB	Upregulated	miRNA-889-3p	JNK/MAPK/ERK pathway	Promotes tumor growth; inhibits apoptosis	[110]
RB	Upregulated	miRNA-25-3p	PTEN	Enhances EMT and cell migration	[111]
RB	Upregulated	miRNA-141-3p	SUSD2	Promotes angiogenesis and proliferation	[112]
RB	Upregulated	miRNA-222	VHL	Promotes chemotherapy resistance	[113]
RB	Upregulated	miRNA-224-3p/miRNA-492	LATS2	Increases cancer invasion	[114,115]
RB	Upregulated	miRNA-92a-3p	KLF2	Enhances tumor-associated angiogenesis	[116]
RB	Downregulated	miRNA-34a/34b-5p	NOTCH signaling	Increases chemosensitivity; suppresses invasion	[117,118]
RB	Downregulated	miRNA-153-3p	IGFR1/Raf/MEK & PI3K/AKT pathways	Leads to unchecked proliferation/survival	[119]
RB	Downregulated	miRNA-361-3p	GLI1/GLI3	Increases expression of Hedgehog factors	[120]
RB	Upregulated	lncRNA-MALAT1	miRNA-20b-5p (STAT3)/miRNA-124 & 598-3p	Upregulates STAT3; chemoresistance	[123,124]
RB	Upregulated	lncRNA-NEAT1	miRNA-124, -3619-5p, -24-3p, -106a, -148-3p	Promotes tumor growth (sponging)	[125,126]
RB	Upregulated	lncRNA-XIST	miRNA-101/miRNA-140-5p & 191-5p	Contributes to EMT, proliferation, and invasion	[127,128]
RB (Hypoxic)	Upregulated	lncRNA-ANRIL	miRNA-328	Contributes to chemotherapeutic resistance	[131]
RB	Downregulated	lncRNAs-MT1JP, -MBLN1, -MEG3	WNT/β-catenin pathway	Tumor suppression	[137,138,139]
RB	Upregulated	Circ-DHDDS	miRNA-361-3p	Promotes RB progression	[142,143]
RB	Upregulated	circ-FAM158A	XIAP, SMAD2, and LRP6	Promotes metastasis and optic nerve invasion	[144,145]
RB	Upregulated	circ-E2F3	miRNA-204-5p (ROCK1)/LASP1	Enhances motility and invasion	[146,147]
RB	Upregulated	circ_RNF20	miRNA-132-3p (PAX6)	Promotes invasion	[148]
RB	Downregulated	circ-TET1	miRNA-492/miRNA-484-3p (WNT/β-catenin)	Targets WNT signaling	[149]
RB	Downregulated	circ-SHPRH	AKT and mTOR	Promotes cell apoptosis	[150]
RB	Downregulated	circ-MKLN1	miRNA-425-5p	Curbs cell invasion	[151]
RB	Downregulated	circ-CUL2	E2F2	Reduces proliferation and migration	[152]
RB	Upregulated	miRNA-320a	TUSC3	Promotes proliferation and apoptotic resistance	[109]
RB	Upregulated	miRNA-889-3p	JNK/MAPK/ERK pathway	Promotes tumor growth; inhibits apoptosis	[110]
RB	Upregulated	miRNA-25-3p	PTEN	Enhances EMT and cell migration	[111]

### 4.3. Rhabdomyosarcoma

Rhabdomyosarcoma (RMS) is a malignant soft tissue cancer that arises from cells of the myogenic lineage [153]. According to the World Health Organization (WHO), there are four main subtypes of RMS based on histological and molecular characteristics [154,155]. These include two commonly diagnosed types, alveolar (ARMS) and embryonal (ERMS), as well as the two rarer subtypes pleomorphic (usually diagnosed in adult patients) and spindle cell/sclerosing RMS, which carries a poor prognosis [156]. Between the two main subtypes, ARMS is less differentiated with a tightly packed alveolar histology and a higher probability of metastasis [157,158]. ARMS-associated fusion genes PAX3-Forkhead box 01 (*FOXO1*) and PAX7-FOXO1, identified in more than half of ARMS patients, drive increased proliferation rates and reduced differentiation [159]. In contrast, ERMS is more differentiated with a round skeletal muscle-like appearance and lacks associated fusion genes, although these have been identified in some rare cell lines [160]. ERMS-associated mutations affect the RAS pathway and have also been identified in tumor protein p53 (*TP53*), BCL6 corepressor (*BCOR*), and/or neurofibromin (*NF1*) [161,162]. In general, most RMS patients are asymptomatic, but localized pain, swelling, and/or impaired movement can present depending on tumor location [163]. Treatment involves a combination of chemotherapy, surgical resection, and radiation depending on tumor type and the extent/site of disease [164,165]. While there have been significant improvements in RMS imaging, diagnostics, and treatment strategies (low to moderate risk survival is 80–90%), patients without complete localized control or with high-risk, metastatic, or recurrent RMS still face survival rates of less than 30% [166]. This highlights a critical need for additional research to improve scientific understanding of RMS and identify novel therapeutic targets (Table 3).

The function and mechanisms of miRNAs in RMS onset and progression are the most studied of all ncRNAs. Compared with other pediatric solid tumor types, RMS-associated miRNA research has slowed in recent years with a focus shift to those involved in muscle growth and development as opposed to pathology. Studies conducted in tumors and cell lines have identified subtype-specific miRNAs. For instance, the ARMS-associated fusion of PAX3-FOXO1 leads to an increase in the expression of miRNAs-486-5p and -9-5p, which increase cell proliferation and invasion [167,168]. The same fusion gene also inhibits the expression of tumor suppressor miRNAs-27a, -221, and -222 [169,170]. miRNA-335-5p is also overexpressed in ARMS, and it may function as a disease biomarker for this subtype [171]. Oncogenic miRNA-130a/b expression is increased in ERMS, leading to increased cell proliferation and decreased levels of peroxisome proliferator-activated receptor gamma (*PPARG*) [172]. Tumor suppressor miRNAs-181a and -212 are both significantly reduced in ERMS leading to reduced myogenic differentiation and increased cell invasion [173]. Other miRNAs have no subtype specification. Oncogenic miRNA-223 is increased pediatric RMS where it promotes inflammation and EMT [174]. Decreased expression of tumor suppressor miRNAs-7 and -324-5p results in elevated alpha-9-integrin (ITGA9) expression and increasing RMS-associated metastasis [175]. Two other tumor suppressor miRNAs, miRNAs-28-3p and -193-5p, are also reduced in both RMS subtypes [176]. miRNAs-223 and -29a/b/c inhibit cancer cell aggressiveness in both ARMS and ERMS [177,178]. Finally, miRNA-26a demonstrates a reduced expression in RMS tissues and is under evaluation as a potential disease biomarker [179].

The role of lncRNAs in RMS remains largely unknown, with only a few RMS-associated molecules identified. lncMYCNOS was studied previously in the pathogenesis of neuroblastoma and in ARMS where tumor growth was inhibited following knockdown [180]. lncRNA-H19 promotes myogenic differentiation through the upregulation of miRNAs-675-3p and -375-5p [181,182]. lncRNAs-SYISL and -NEAT1 both reduce myogenic differentiation and drive accelerated proliferation rates of RMS cells [183,184]. Inhibition of tumor suppressor lncRNA-GAS5 leads to increased proliferation and decreased apoptosis [185]. Finally, linc-MD1 decreases myogenic differentiation and enhances proliferation via binding inhibition miRNAs-206 and -133b [186].

Similar to lncRNAs, the expression and mechanisms underlying circRNAs in RMS pathophysiology are poorly understood. There are only three RMS-associated circRNAs that have been studied for disease relevance and mechanism. circ-AFF1 is increased in both ARMS and ERMS, where it increases cell migration via altered cellular adhesion [187]. Overexpression of circ-VAMP3 and circ-ZNF609 in RMS disrupts normal cell cycle regulation in ARMS and alters AKT pathway signaling [188,189]. The lack of knowledge regarding circRNAs in RMS pathology highlights a critical area that could benefit from further study to better understand the roles of these molecules in the disease and identify potential target for therapy development.

**Table 3:** Experimentally validated ncRNAs known to contribute to the progression and metastatic potential of RMS.

Disease/ Subgroup	Expression	Non-Coding RNA	Validated Target Gene(s)	Functional Role	Reference(s)
ARMS	Upregulated	miRNA-486-5p/-9-5p	PAX3-FOXO1 induced	Increases proliferation and invasion	[167,168]
ARMS	Downregulated	miRNA-27a/-221/-222	PAX3-FOXO1 repressed	Tumor suppression (loss of)	[169,170]
ERMS	Upregulated	miRNA-130a/b	PPARG	Increases proliferation	[172]
ERMS	Downregulated	miRNA-181a/-212	N/A	Reduced myogenic differentiation; increased invasion	[173]
RMS (Both)	Downregulated	miRNA-7/-324-5p	ITGA9	Increases metastasis	[175]
RMS (Both)	Upregulated	lncMYCNOS	N/A	Promotes tumor growth	[180]
RMS (Both)	Upregulated	lncRNA-H19	miRNA-675-3p/-375-5p	Promotes myogenic differentiation	[181,182]
RMS (Both)	Upregulated	lncRNA-SYISL/ NEAT1	N/A	Reduces differentiation; accelerates proliferation	[183,184]
RMS (Both)	Upregulated	linc-MD1	miRNA-206/-133b	Decreases differentiation; enhances proliferation	[186]
RMS (Both)	Upregulated	circ-AFF1	Cellular adhesion	Increases cell migration	[187]
ARMS	Upregulated	circ-VAMP3/circ-ZNF609	AKT pathway	Disrupts cell cycle regulation	[188,189]

### 4.4. Hepatoblastoma

Hepatoblastoma (HB) is a rare, malignant liver tumor affecting children three years of age and younger [190]. Contributing risk factors include overgrowth syndromes or congenital syndromes such as Beckwith–Wiedemann syndrome (BWS) and familial adenomatous polyposis (FAP) due to chromosomal alterations and inherited mutations in adenomatous polyposis colic (APC) gene structure [191,192]. HB diagnoses also demonstrate significantly higher rates in Asian countries than Western counterparts [193]. Also, premature children with low birth weight (<1500 g) demonstrate predisposition for the disease [194]. Genetic studies have identified oncogenic mutations in the beta-catenin (*CTNNB1*) and NFE2 like BZIP transcription factor 2 (*NFE2L2*) genes, as well as the promoter regions of telomerase reverse transcriptase (*TERT*) that result in increased tumor growth [195,196]. Symptoms at clinical presentation include a painless abdominal mass (often distended), jaundice, unexplained weight loss, and/or hepatomegaly [190]. Official diagnosis is confirmed with a combination of imaging (CT or MRI), serum levels of HB biomarker alpha fetoprotein (AFP), and/or histology following biopsy [197]. Treatment is multimodal and includes a combination of surgical resection and adjuvant/neoadjuvant chemotherapy, with liver transplant utilized in a large portion of higher risk, therapy responsive cases [198,199]. HB patients treated with the standard of care see overall survival rates around 70%, but patients with small cell undifferentiated, metastatic, or relapsed disease have an unfavorable prognosis [200]. Next-generation sequencing of HB cell lines and patient-derived tissues have improved our understanding of the role of ncRNAs in HB pathogenesis, progression, and relapse (Table 4).

The role of miRNAs in the pathogenesis of HB is the most well-characterized of all the ncRNAs, although knowledge remains moderately sparse. Due to the importance of the WNT pathway in HB disease progression, several WNT pathway-associated miRNAs have been identified, including miRNAs-193b, -760, and -23a-5p [201,202]. The PI3K/AKT/mTOR pathway is also dysregulated in HB, with miRNAs-193a-5p, -495, and -206 showing oncogenic dysregulation in HB cells [203,204]. miRNAs have also shown promise as potential biomarkers with clinical importance, including: miRNAs-17, -21, -19b, -146a, -492, and -186 [205,206,207,208]. More recently, studies of miRNA variance between HB tumors and normal liver have begun to unravel some of the geographical differences observed in HB prevalence. Polymorphisms in miR-34b/c (rs4938723) have been described that alter HB susceptibility in an Eastern Chinese population of patients, adding geographical data to biomarker and prognostic knowledge [209].

Oncogenic alterations in lncRNA also contribute to HB risk and malignancy. For instance, upregulation of lncRNAs MIR205HG, neighbor of BRCA1 lncRNA2 (NBR2), taurine upregulated 1 (TUG1), HOXA-AS2, ZFAS1, and lnc01124 have been detected in HB samples, where they modify cancer cell stemness to enhance invasion and proliferation via MAPK and PI3K/AKT signaling pathways [210,211,212,213]. Similarly, upregulation of lncRNA OIP5-AS1 promotes HB invasion via the WNT/β-catenin pathway [214]. Other lncRNAs that have shown associations with increased HB malignancy and EMT are HOXA-AS2, ZFAS1, and CRNDE [214,215]. Finally, geographic polymorphisms in lncRNA-H19 that correlate to an increased risk of disease have also been identified [216].

circRNAs have shown clinical relevance in adult liver cancers, and recent studies have begun to shed light on their roles in pediatric HB [217]. circ_0015756 is upregulated in HB patient samples and cell lines, where it enhances HB proliferation and viability [218]. Similarly, circ_0000594 promotes HB malignancy via binding suppression of miRNA-217 [219]. circHMGCS1 (circ_0072391) functions as a sponge for tumor suppressor miR-503-5p, resulting in abnormal activation of the IGF/PI3K/Akt pathway and oncogenic alterations in glutamine metabolism [220]. Similarly, circRNA_CCT2 has pro-tumor properties via activation of the WNT/β-catenin pathway [221]. Several circRNAs have shown potential for halting HB progression, such as circMTO1, circEPB41L2, and circDLC1 [222,223,224]. Finally, circRNA_SORE has been linked to increased chemoresistance in HB [225].

**Table 4:** Experimentally validated ncRNAs with known role in HB progression, metastasis, and EMT.

Disease/ Subgroup	Expression	Non-Coding RNA	Validated Target Gene(s)	Functional Role	Reference(s)
HB	Upregulated	miRNA-193b, -760, -23a-5p	WNT pathway	Promotes disease progression	[201,202]
HB	Upregulated	miRNA-193a-5p, -495, -206	PI3K/AKT/mTOR pathway	Promotes oncogenesis	[203,204]
HB	Upregulated	lncRNA MIR205HG, NBR2, TUG1, HOXA-AS2, ZFAS1, lnc01124	MAPK and PI3K/AKT pathways	Modifies stemness; enhances invasion and proliferation	[210,211,212,213]
HB	Upregulated	lncRNA OIP5-AS1	WNT/β-catenin pathway	Promotes invasion	[214]
HB	Upregulated	lncRNA HOXA-AS2, ZFAS1, CRNDE	EMT (process)	Increases malignancy and EMT	[215,216]
HB	Upregulated	circ_0015756	N/A	Enhances proliferation and viability	[219]
HB	Upregulated	circ_0000594	miRNA-217	Promotes malignancy	[220]
HB	Upregulated	circHMGCS1 (circ_0072391)	miR-503-5p (IGF/PI3K/Akt)	Activates IGF/PI3K/Akt; alters glutamine metabolism	[221]
HB	Upregulated	circRNA_CCT2	WNT/β-catenin pathway	Pro-tumor properties	[222]
HB	Downregulated/Suppressive	circMTO1, circEPB41L2, circDLC1	N/A	Halts HB progression	[223,224,225]
HB	Upregulated	circRNA_SORE	N/A	Linked to increased chemoresistance	[226]
HB	Upregulated	miRNA-193b, -760, -23a-5p	WNT pathway	Promotes disease progression	[201,202]

### 4.5. Osteosarcoma

Osteosarcoma (OS) is the most common primary bone tumor in children and adolescents. The disease demonstrates a bimodal age distribution, occurring between 10 and 14 years and in those over 65 years of age [226,227]. OS can arise in any bone, but it most commonly affects the metaphyses of lower limb bones [228]. Patients often present with persistent localized pain and/or swelling that cannot be attributed to accident or injury [229]. Treatment is multimodal, involving surgical excision and neoadjuvant/adjuvant chemotherapy with methotrexate, adriamycin (doxorubicin), and platinum (cisplatin; MAP therapy) [230,231]. MAP implementation and surgical advances have improved overall survival to over 80%, but patients with metastatic OS, relapsed OS, or with poor chemotherapeutic response still face survival rates of 30% or below [232,233]. Therapeutic development has been significantly hampered by the fact that OS demonstrates a high degree of genetic heterogeneity, and only a few OS-associated germline mutations have been identified [234]. These include mutations in *TP53*, MYC proto-oncogene, *RB1*, isocitrate dehydrogenase 1 (*IDH1*), and cyclin-dependent kinase inhibitor 2A (*CDKN2A*) [235,236,237,238,239]. Because of this, OS-related ncRNA research has focused on identifying molecules associated with progression, therapy resistance, and for use as potential biomarkers (Table 5).

As with many of the other pediatric cancers discussed in this review, miRNAs are the most thoroughly studied OS-associated ncRNA. Studies have characterized miRNA biomarkers, oncogenic and tumor suppressive miRNAs, and elucidated many of the target genes, biological functions, and underlying molecular signaling pathways. These miRNAs have been the focus of several excellent recent reviews and will not be discussed in depth here [240,241].

lncRNAs play critical roles in the modulation of OS transcription patterns, post-transcriptional modifications, mRNA stabilization, and epigenetic regulation [242]. Downregulation of tumor suppressor lncRNA-HIF2PUT correlates with prognosis in OS cell lines and tumors via regulation of hypoxia-inducible factor 2 (*HIF2*) [243,244]. lncRNA-MALAT1 increases OS cell proliferation and metastasis through PI3K/Akt overactivation and the inhibition of high mobility group box 1 (*HMGB1)* [245]. Similarly, lncRNA-HOTAIR promotes OS pathogenesis through Akt/mTOR pathway activation, increased zinc finger E-box bonding homeobox 1 (*ZEB1*), and the inhibition of mineralization via bone-specific alkaline phosphatase (ALP) activity [246,247]. In OS, lncRNA-DANCR expression is correlated with disease stage, enhances stemness and improves immune evasion through the upregulation of the tyrosine kinase AXL and enhanced Musashi RNA binding protein 2 (*MSI2*) expression [248,249]. The lncRNA-CRNDE demonstrates diagnostic and prognostic expression patterns, and promotes metastasis, EMT, and enhances proliferation through overactivation of the WNT/β-catenin, ATP binding cassette subfamily C member 12 (*ABCC12/MRP9*), and NOTCH1 signaling pathways [250,251,252,253]. lncRNA-SNHG12 promotes OS metastasis, contributes to doxorubicin resistance, and demonstrates clinical trends that suggest its potential use as a diagnostic tool [254,255]. Enhanced expression of lncRNA-UCA1 correlates with prognosis and with increased invasion and tumor growth [256,257]. Finally, lncRNA-THOR promotes OS tumor growth via the overactivation of the Akt/ERK pathway and by the increased stability of SRY-box transcription factor 9 (*SOX9*) mRNA [258].

circRNAs have demonstrated biological functions in both adult and pediatric OS, where they alter cell–cell communication, gene and protein expression, therapy response, and the physical and biochemical composition of the TME [259]. circ-FOXM1 (hsa_circ_0025033) sponges the tumor suppressor miRNAs-320a and -320b which contributes to the proliferation and cell migration via WNT pathway activation [260]. Similarly, circRAB3IP (hsa_circRNA_0000419) promotes invasion by sponging the tumor suppressor miRNA-580-3p to enhance the expression of twist family BHLH transcription factor (*TWIST1*) [261]. Upregulation of circ_03955 enhances metadherin (*MTDH*) expression through binding inhibition of miRNA-3662 [262]. circ-LRP6 promotes invasion through the regulation of histone deacetylase 4 (*HDAC4*) and *HMGB1* [263,264]. OS-associated upregulation of circDOCK1 (hsa_circ_0020378) enhances apoptotic resistance and chemotherapy resistance by enhancing *IGF-1R*, DNA methyltransferases 3A (*DNMT3A*), and lymphoid enhancer binding factor 1 (*LEF1*) expression [265,266,267]. The expression of circ_001422 correlates with stage and extent of disease (primary versus metastatic), and promotes activation of the fibroblast growth factor 2 (FGF2)/PI3K/Akt pathway and downregulation of cell cycle regulator E2F transcription factor 3 (*E2F3*) [268,269]. More recently, oncogenic circPVT1 (hsa_circ_0001821) has shown diagnostic promise via roles in multiple oncogenic pathways [270,271].

**Table 5:** Experimentally validated ncRNAs known to play roles in the disease prognosis, progression, therapy resistance, and invasion of OS.

Disease/ Subgroup	Expression	Non-Coding RNA	Validated Target Gene(s)	Functional Role	Reference(s)
OS	Down-regulated	lncRNA-HIF2PUT	HIF2	Correlates with poor prognosis	[244,245]
OS	Upregulated	lncRNA-MALAT1	PI3K/Akt/HMGB1	Increases proliferation and metastasis	[246]
OS	Upregulated	lncRNA-HOTAIR	Akt/mTOR/ZEB1/ALP	Promotes pathogenesis; inhibits mineralization	[247,248]
OS	Upregulated	lncRNA-DANCR	AXL/MSI2	Enhances stemness and immune evasion	[249,250]
OS	Upregulated	lncRNA-CRNDE	WNT/β-catenin/ABCC12/NOTCH1	Promotes metastasis, EMT, and proliferation	[251,252,253,254]
OS	Upregulated	lncRNA-SNHG12	N/A	Promotes metastasis; doxorubicin resistance	[255,256]
OS	Upregulated	lncRNA-UCA1	N/A	Increases invasion and tumor growth	[257,258]
OS	Upregulated	lncRNA-THOR	Akt/ERK/SOX9	Promotes tumor growth; stabilizes mRNA	[259,260]
OS	Upregulated	circ-FOXM1 (hsa_circ_0025033)	miRNA-320a/b/WNT pathway	Contributes to proliferation and migration	[262]
OS	Upregulated	circRAB3IP (hsa_circ_0000419)	miRNA-580-3p/TWIST1	Promotes invasion	[263]
OS	Upregulated	circ_03955	miRNA-3662/MTDH	Enhances metadherin expression	[264]
OS	Upregulated	circ-LRP6	HDAC4/HMGB1	Promotes invasion	[265,266]
OS	Upregulated	circDOCK1 (hsa_circ_0020378)	IGF-1R/DNMT3A/LEF1	Apoptotic and chemotherapy resistance	[267,268,269]
OS	Upregulated	circ_001422	FGF2/PI3K/Akt/E2F3	Promotes activation of growth pathways	[270,271]
OS	Upregulated	circPVT1 (hsa_circ_0001821)	Multiple oncogenic pathways	Diagnostic potential	[272,273]
OS	Down-regulated	lncRNA-HIF2PUT	HIF2	Correlates with poor prognosis	[244,245]

### 4.6. Ewing Sarcoma

Ewing sarcoma (ES) is a rare, aggressive bone and soft tissue cancer that arises in cells of the bone-derived mesenchymal stem cell (MSCs) lineage in children and adolescents [272,273]. Genetic analyses have uncovered ES-associated chromosomal translocations such as EWS RNA binding protein 1 (*EWSR1*) with friend leukemia integration 1 (*FLI1*; ~85% of cases) and ETS-related gene (*ERG*), ETS translocation variant 1 (*ETV1*), ETS variant gene 4 (E1AF) or FEV transcription factor (*FEV*), which together account for 10–15% of cases [274,275,276]. Common symptoms include fatigue, unexpected weight loss, bone pain or fracture, localized pain, or detection of a palpable mass [277]. Diagnosis of localized disease occurs in approximately 75% of cases, but 25% of cases are diagnosed after the development of bone marrow or pulmonary metastases [272]. Multimodal treatment is utilized depending on the disease stage and location, combining neoadjuvant/adjuvant chemotherapy, radiotherapy, and surgical resection [278,279]. Survival rates for patients diagnosed with localized disease are around 70%, but the survival rates significantly drop (~30%) in those diagnosed with metastatic and/or relapsed disease [280,281]. ncRNAs are being explored for their potential roles as biomarkers and for therapeutic development (Table 6).

Biological functions, mechanisms, and clinical relevance of ES-associated miRNAs have been examined in both patient tumors and cell lines. miRNAs-20b, and -34b both demonstrate correlations with ES disease progression [282]. miRNAs-30d, -125b, and -21, both overexpressed in ES, enhance the expression of Bcl-2-like protein 4 (*BAX*) to prevent cancer cell apoptosis [283,284,285]. miRNA-181c promotes tumor growth via suppression of tumor necrosis factor receptor super family member-6 (*TNFRSF6*) [286]. Enhanced expression of miRNA-130b contributes to cell cycle dysregulation by through activation of cell division cycle 42 (*CDC42*) [287]. Conversely, miRNA-683 has been shown to inhibit angiogenesis by targeting *VEGFA* mRNA for degradation [288]. The let7 family of tumor-suppressor miRNAs also plays a role in ES [289]. Let-7a forms RISCs withEWSR1 mRNA, which inhibits protein production [290]. Let-7b targets the RAS pathway to inhibit tumor cell survival and also reduces the expression of HIF1α within the TME [291]. miRNA-708-5p has been shown to inhibit pathogenic ECM remodeling by targeting matrix metallopeptidase 12 (MMP12), and miRNA-27a reduces cell proliferation and tumor growth by targeting IGF-1) [292,293].

The studies of lncRNAs in ES pathogenesis are still fairly limited and confined primarily to in vitro studies of ES cell models. However, a recent study examining RNA-seq data from patient samples detected significant upregulation in expression patterns of several lncRNAs (SNHG17, LINC00623, WAC-AS1, SSBP3-AS1, and TDRG1) that are indicative of poor overall survival rates [294]. Similarly, microarray analyses have identified ES-associated increases in lncRNAs PncCCND1_B and FOXP4-ASI expression, and have demonstrated these target cyclin D1 (*CCND1*) and thymopoietin (*TMPO*), respectively via miRNA sponging [295,296]. HOTAIR and TUG1 are upregulated in ES (as in other pediatric solid tumors discussed here) and contribute to disease progression [297,298]. lncRNA METTL3 promotes cell migration through competitive binding interactions with miRNA-124-3p, which results in elevated cyclin-dependent kinase 4 (*CDK4*) expression in tumor-derived cells [299]. Finally, studies of genetic variations in patient samples uncovered polymorphisms in lncRNA SENCR, associated with FLI1, and their role in the regulation of ES-associated upregulation of insulin-like growth factor 2 mRNA-binding protein 3 (*IGF2BP3*) [300].

The role of circRNAs in ES remains mostly unexplored. To date, only one circRNA has been studied in ES pathogenesis. circZNF609 has been shown to be associated with the EWS-FLI1 gene fusion and to promote metastasis and inhibit apoptosis in ES cell lines through sponging interaction with miRNA-145-5p [301]. Future studies into the role of circRNAs in ES will be beneficial in identifying novel targets and biomarkers to improve diagnosis and treatment.

**Table 6:** Experimentally validated ncRNAs known with roles in ES-associated cell cycle regulation, apoptosis, ECM remodeling, and angiogenesis.

Disease/ Subgroup	Expression	Non-Coding RNA	Validated Target Gene(s)	Functional Role	Reference(s)
ES	Upregulated	miRNAs-30d, -125b, -21	BAX	Prevents cancer cell apoptosis	[284,285,286]
ES	Upregulated	miRNA-181c	TNFRSF6	Promotes tumor growth	[287]
ES	Upregulated	miRNA-130b	CDC42	Cell cycle dysregulation	[288]
ES	Downregulated	miRNA-683	VEGFA	Inhibits angiogenesis	[289]
ES	Downregulated	Let-7a	EWSR1	Inhibits protein production (RISC)	[291]
ES	Downregulated	Let-7b	RAS pathway/HIF1α	Inhibits survival; reduces TME hypoxia	[292]
ES	Downregulated	miRNA-708-5p	MMP12	Inhibits pathogenic ECM remodeling	[293]
ES	Downregulated	miRNA-27a	IGF-1	Reduces proliferation and tumor growth	[294]
ES	Upregulated	lncRNA PncCCND1_B	CCND1 (Cyclin D1)	Promotes progression (sponging)	[296]
ES	Upregulated	lncRNA FOXP4-ASI	TMPO (Thymopoietin)	Promotes progression (sponging)	[297]
ES	Upregulated	lncRNA METTL3	miRNA-124-3p/CDK4	Promotes cell migration	[300]
ES	Polymorphism	lncRNA SENCR	FLI1/IGF2BP3	Upregulates IGF2BP3	[301]
ES	Upregulated	circZNF609	miRNA-145-5p/EWS-FLI1	Promotes metastasis; inhibits apoptosis	[302]

## 5. Discussion

Cancer is a multifactorial disease that affects both adult and pediatric patients. Improvements in diagnostics, imaging, and therapeutic techniques have greatly improved overall survival and event free survival rates for many cancers, but recurrence and therapy resistance still comprise a significant number of cases. Towards the goal of finding novel therapeutic strategies, research has turned to determining the role of epigenetic mechanisms such as aberrant DNA methylation, histone modifications, and non-coding RNAs [302].

The term non-coding RNAs encompasses a wide range of biological molecules that have been shown to play vital roles in health and disease [303]. In the field of pediatric medicine, genome and tumor RNA-sequencing have greatly improved our knowledge of prognostic biomarkers and the mechanistic roles of miRNAs, lncRNAs, and circRNAs in the biology of many solid cancers. These findings have led to the development and incorporation of targeted therapies currently approved or in clinical trials, such as RAF and/or MEK inhibitors (dabrafenib, trametinib, and selumetinib) in the treatment of low-grade gliomas (LGGs) and some gene-mutant solid tumors to augment traditional systemic treatment [304,305]. However, while knowledge of cancer-associated ncRNA variances has greatly improved, the implementation of non-coding RNA-targeted therapies is still hampered by off-target effects, drug toxicity, and the existing knowledge gaps regarding the functions and disease/stage specificities of ncRNAs.

## 6. Conclusions

This review highlights current knowledge regarding the roles and underlying mechanisms of ncRNAs in many pediatric solid cancers in addition to critical knowledge gaps where the role of these molecules remains poorly understood. Also, differences in ncRNA profiles between disease subtypes, identification of alterations upon relapse, and primary versus metastatic disease remain unknown. Further research into these areas would greatly improve biomarker and therapeutic target identification to improve survival rates and patient quality of life.

## Data Availability

No new data were created or analyzed in this study. Data sharing is not applicable to this article.

## Abbreviations

The following abbreviations are used in this manuscript:

ncRNA	Non-coding RNA
miRNA	MicroRNA
lncRNA	Long non-coding RNA
circRNA	Circular RNA
CNS	Central nervous system
NGS	Next generation sequencing
snoRNA	Small nucleolar RNA
piRNA	Piwi-interacting RNA
siRNA	Small interfering RNA
scRNA	Small cytoplasmic RNA
RISC	RNA-induced silencing complex
UTR	Untranslated region
shRNA	Short-hairpin RNA
PolI	RNA polymerase I
PolII	RNA polymerase II
PolIII	RNA polymerase III
TRBP	Transactivation response RNA binding protein
EIcircRNA	Exon-Intron circRNA
ciRNA	Intron-only circRNA
ecircRNA	Exon-only circRNA
MB	Medulloblastoma
SHH	Sonic-Hedgehog
WNT	Wingless-related integration site
NRP2	Neuropilin 2
VEGFA	Vascular endothelial growth factor alpha
FOXM1	Forkhead box M1
RB	Retinoblastoma
RB1	Retinoblastoma 1 gene
MYCN	MYCN Proto-oncogene
TUSC3	Tumor suppressor candidate 3
JNK	C-Jun N-terminal kinase
MAPK	Mitogen-activated protein kinase (MAPK)
ERK	Extracellular signal-related kinase
EMT	Epithelial-to-mesenchymal transition
SUDS2	Sushi domain-containing protein 2
VHL	Von Hippel-Lindau
KLF2	Krüppel-like factor 2
NOTCH	Neurogenic locus notch homology protein
IGFR1	Insulin-like growth factor 1 receptor
RAS	Rapidly accelerated fibrosarcoma
MEK	Mitogen-activated protein kinase kinase 1
PI3K	Phosphoinositide 3-kinase
AKT	AKT serine/threonine kinase 1
GLI1	GLI family zinc finger 1
GLI3	GLI family zinc finger 3
PIK3CA	Phosphatidylinositol-4,5-bisphosphate 3-kinase catalytic subunit alpha
mTOR	Mammalian target of rapamycin
AEG-1	Astrocyte elevated gene 1
PAX6	Paired box 6
ABCB1	Multidrug resistance protein 1
XIAP	X-linked inhibitor of apoptosis
WIF1	WNT inhibitory factor 1
TME	Tumor microenvironment
SMAD2	Smad family member 2
LRP6	Lipoprotein receptor-related protein 6
E2F2	E2F transcription factor 2
RMS	Rhabdomyosarcoma
ARMS	Alveolar rhabdomyosarcoma
ERMS	Embryonal rhabdomyosarcoma
FOX01	Forkhead box 01
TP53	Tumor protein p53
BCOR	BCL6 corepressor
NF1	Neurofibromin
PPARG	Peroxisome proliferator-activated receptor gamma
ITGA9	Alpha-9-integrin
SNAIL	Snail family transcriptional repressor 1
MyoD	Myoblast determination protein 1
HB	Hepatoblastoma
BWS	Beckwith–Wiedemann syndrome
FAP	Familial adenomatous polyposis
APC	Adenomatous polyposis colic
CTNNB1	Beta-catenin gene
NFE2L2	NFE2 like BZIP transcription factor 2
TERT	Telomerase reverse transcriptase
AFP	Alpha fetoprotein
OS	Osteosarcoma
MAP	Methotrexate, adriamycin (doxorubicin), and platinum therapy
CDKN2A	Cyclin-dependent kinase inhibitor 2A
HIF2	Hypoxia inducible factor 2
ZEB1	Zinc finger E-box bonding homeobox 1
ALP	Alkaline phosphatase
MSI2	Musashi RNA binding protein 2
SOX9	SRY-box transcription factor 9
TWIST1	Twist family BHLH transcription factor
MTDH	Metadherin
HDAC4	Histone deacetylase 4
HMGB1	High mobility group box 1
DNMT3A	DNA methyltransferases 3A
LEF1	Lymphoid enhancer binding factor 1
FGF2	Fibroblast growth factor 2
E2F3	E2F transcription factor 3
IDH1	Isocitrate dehydrogenase 1
IDH2	Isocitrate dehydrogenase 2
TIMP3	TIMP metallopeptidase 3
FSCN1	Fascin-1
VEGFC	Vascular endothelial growth C
EZH2	Enhancer of Zeste homolog 2
KLF6	KLF transcription factor 6
LGG	Low-grade glioma
CCND1	Cyclin D1
IGF2BP3	Insulin-like growth factor 2 mRNA-binding protein 3
CDC42	Cell division cycle 42
TNFRSF6	Tumor necrosis factor receptor super family member-6
FEV	FEV transcription factor
E1AF	ETS variant gene 4
FLI1	Friend leukemia integration 1
ERG	ETS-related gene
ETV1	ETS translocation variant 1
EWSR1	EWS RNA binding protein 1

## References

[1] Bizuayehu H.M., Ahmed K.Y., Kibret G.D., Dadi A.F., Belachew S.A., Bagade T., Tegegne T.K., Venchiarutti R.L., Kibret K.T., Hailegebireal A.H. (2024). Global disparities of cancer and its projected burden in 2050. JAMA Netw. Open.

[2] Miller K.D., Nogueira L., Devasia T., Mariotto A.B., Yabroff K.R., Jemal A., Kramer J., Siegel R.L. (2022). Cancer treatment and survivorship statistics, 2022. CA Cancer J. Clin..

[3] Byrne S., Boyle T., Ahmed M., Lee S.H., Benyamin B., Hyppönen E. (2023). Lifestyle, genetic risk and incidence of cancer: A prospective cohort study of 13 cancer types. Int. J. Epidemiol..

[4] Novikov N.M., Zolotaryova S.Y., Gautreau A.M., Denisov E.V. (2021). Mutational drivers of cancer cell migration and invasion. Br. J. Cancer.

[5] Derks L.L., van Boxtel R. (2023). Stem cell mutations, associated cancer risk, and consequences for regenerative medicine. Cell Stem Cell.

[6] LaHaye S., Fitch J.R., Voytovich K.J., Herman A.C., Kelly B.J., Lammi G.E., Arbesfeld J.A., Wijeratne S., Franklin S.J., Schieffer K.M. (2021). Discovery of clinically relevant fusions in pediatric cancer. BMC Genom..

[7] Mella C., Tsarouhas P., Brockwell M., Ball H.C. (2025). The Role of Chronic Inflammation in Pediatric Cancer. Cancers.

[8] Siegel D.A., King J.B., Lupo P.J., Durbin E.B., Tai E., Mills K., Van Dyne E., Buchanan Lunsford N., Henley S.J., Wilson R.J. (2023). Counts, incidence rates, and trends of pediatric cancer in the United States, 2003–2019. JNCI J. Natl. Cancer Inst..

[9] Siegel D.A., Richardson L.C., Henley S.J., Wilson R.J., Dowling N.F., Weir H.K., Tai E.W., Buchanan Lunsford N. (2020). Pediatric cancer mortality and survival in the United States, 2001–2016. Cancer.

[10] Ferrari A., Brennan B., Casanova M., Corradini N., Berlanga P., Schoot R.A., Ramirez-Villar G.L., Safwat A., Guillen Burrieza G., Dall’Igna P. (2022). Pediatric non-rhabdomyosarcoma soft tissue sarcomas: Standard of care and treatment recommendations from the European Paediatric Soft Tissue Sarcoma Study Group (EpSSG). Cancer Manag. Res..

[11] Trubicka J., Grajkowska W., Dembowska-Bagińska B. (2022). Molecular markers of pediatric solid tumors—Diagnosis, optimizing treatments, and determining susceptibility: Current state and future directions. Cells.

[12] Effinger K., Haardörfer R., Marchak J.G., Escoffery C., Landier W., Kommajosula A., Hendershot E., Sadak K., Eshelman-Kent D., Kinahan K. (2023). Current pediatric cancer survivorship practices: A report from the Children’s Oncology Group. J. Cancer Surviv..

[13] Mobley E.M., Moke D.J., Milam J., Ochoa-Dominguez C.Y., Stal J., Mitchell H., Aminzadeh N., Bolshakova M., Mailhot Vega R.B., Dinalo J. (2023). Disparities in pediatric cancer survivorship care: A systematic review. Cancer Med..

[14] Chen B., Dragomir M.P., Yang C., Li Q., Horst D., Calin G.A. (2022). Targeting non-coding RNAs to overcome cancer therapy resistance. Signal Transduct. Target. Ther..

[15] Ferrer J., Dimitrova N. (2024). Transcription regulation by long non-coding RNAs: Mechanisms and disease relevance. Nat. Rev. Mol. Cell Biol..

[16] Lin X., Lu Y., Zhang C., Cui Q., Tang Y.-D., Ji X., Cui C. (2024). LncRNADisease v3. 0: An updated database of long non-coding RNA-associated diseases. Nucleic Acids Res..

[17] Statello L., Guo C.-J., Chen L.-L., Huarte M. (2021). Gene regulation by long non-coding RNAs and its biological functions. Nat. Rev. Mol. Cell Biol..

[18] Mattick J.S., Amaral P.P., Carninci P., Carpenter S., Chang H.Y., Chen L.-L., Chen R., Dean C., Dinger M.E., Fitzgerald K.A. (2023). Long non-coding RNAs: Definitions, functions, challenges and recommendations. Nat. Rev. Mol. Cell Biol..

[19] Loganathan T., Doss C G.P. (2023). Non-coding RNAs in human health and disease: Potential function as biomarkers and therapeutic targets. Funct. Integr. Genom..

[20] Kirstein N., Dokaneheifard S., Cingaram P.R., Valencia M.G., Beckedorff F., Gomes Dos Santos H., Blumenthal E., Tayari M.M., Gaidosh G.S., Shiekhattar R. (2023). The Integrator complex regulates microRNA abundance through RISC loading. Sci. Adv..

[21] Sarkar N., Kumar A. (2025). Paradigm shift: microRNAs interact with target gene promoters to cause transcriptional gene activation or silencing. Exp. Cell Res..

[22] Vishnoi A., Rani S. (2022). miRNA biogenesis and regulation of diseases: An updated overview. MicroRNA Profiling Methods Protoc..

[23] Gharehzadehshirazi A., Zarejousheghani M., Falahi S., Joseph Y., Rahimi P. (2023). Biomarkers and corresponding biosensors for childhood cancer diagnostics. Sensors.

[24] Prieto-Colomina A., Fernández V., Chinnappa K., Borrell V. (2021). MiRNAs in early brain development and pediatric cancer: At the intersection between healthy and diseased embryonic development. Bioessays.

[25] Esperanza-Cebollada E., Gómez-González S., Perez-Jaume S., Vega-García N., Vicente-Garcés C., Richarte-Franqués M., Rives S., Català A., Torrebadell M., Camós M. (2023). A miRNA signature related to stemness identifies high-risk patients in paediatric acute myeloid leukaemia. Br. J. Haematol..

[26] Iyer M.K., Niknafs Y.S., Malik R., Singhal U., Sahu A., Hosono Y., Barrette T.R., Prensner J.R., Evans J.R., Zhao S. (2015). The landscape of long noncoding RNAs in the human transcriptome. Nat. Genet..

[27] Bjørklund S.S., Aure M.R., Häkkinen J., Vallon-Christersson J., Kumar S., Evensen K.B., Fleischer T., Tost J., Sahlberg K.K. (2022). Subtype and cell type specific expression of lncRNAs provide insight into breast cancer. Commun. Biol..

[28] Chen L.-L. (2022). Towards higher-resolution and in vivo understanding of lncRNA biogenesis and function. Nat. Methods.

[29] Shah I.M., Dar M.A., Bhat K.A., Dar T.A., Ahmad F., Ahmad S.M. (2022). Long non-coding RNAs: Biogenesis, mechanism of action and role in different biological and pathological processes. Recent Advances in Noncoding RNAs.

[30] Illarregi U., Lopez-Lopez E. (2024). LncRNA expression and regulatory networks across pediatric cancers. Transl. Pediatr..

[31] Liu F., Xiong Q.-W., Wang J.-H., Peng W.-X. (2023). Roles of lncRNAs in childhood cancer: Current landscape and future perspectives. Front. Oncol..

[32] Yi Q., Feng J., Lan W., Shi H., Sun W., Sun W. (2024). CircRNA and lncRNA-encoded peptide in diseases, an update review. Mol. Cancer.

[33] Yang Y., Zhong Y., Chen L. (2025). EIciRNAs in focus: Current understanding and future perspectives. RNA Biol..

[34] Chen J., Gu J., Tang M., Liao Z., Tang R., Zhou L., Su M., Jiang J., Hu Y., Chen Y. (2022). Regulation of cancer progression by circRNA and functional proteins. J. Cell. Physiol..

[35] Xu F., Xiao Q., Du W.W., Wang S., Yang B.B. (2024). CircRNA: Functions, applications and prospects. Biomolecules.

[36] Pisignano G., Michael D.C., Visal T.H., Pirlog R., Ladomery M., Calin G.A. (2023). Going circular: History, present, and future of circRNAs in cancer. Oncogene.

[37] Di Timoteo G., Rossi F., Bozzoni I. (2020). Circular RNAs in cell differentiation and development. Development.

[38] Alcantara J.H., Ornos E.D.B., Tantengco O.A.G. (2023). Global trends, gaps, and future agenda in medulloblastoma research: A bibliometric analysis. Child’s Nerv. Syst..

[39] Northcott P.A., Buchhalter I., Morrissy A.S., Hovestadt V., Weischenfeldt J., Ehrenberger T., Gröbner S., Segura-Wang M., Zichner T., Rudneva V.A. (2017). The whole-genome landscape of medulloblastoma subtypes. Nature.

[40] Abeysundara N., Rasnitsyn A., Fong V., Bahcheli A., Van Ommeren R., Juraschka K., Vladoiu M., Ong W., Livingston B., de Antonellis P. (2025). Metastatic medulloblastoma remodels the local leptomeningeal microenvironment to promote further metastatic colonization and growth. Nat. Cell Biol..

[41] Choi J.Y. (2023). Medulloblastoma: Current perspectives and recent advances. Brain Tumor Res. Treat..

[42] Cotter J.A., Hawkins C. (2022). Medulloblastoma: WHO 2021 and beyond. Pediatr. Dev. Pathol..

[43] Funakoshi Y., Sugihara Y., Uneda A., Nakashima T., Suzuki H. (2023). Recent advances in the molecular understanding of medulloblastoma. Cancer Sci..

[44] Fang F.Y., Rosenblum J.S., Ho W.S., Heiss J.D. (2022). New developments in the pathogenesis, therapeutic targeting, and treatment of pediatric medulloblastoma. Cancers.

[45] Vinchon M., Leblond P. (2021). Medulloblastoma: Clinical presentation. Neurochirurgie.

[46] Louis D.N., Perry A., Reifenberger G., Von Deimling A., Figarella-Branger D., Cavenee W.K., Ohgaki H., Wiestler O.D., Kleihues P., Ellison D.W. (2016). The 2016 World Health Organization classification of tumors of the central nervous system: A summary. Acta Neuropathol..

[47] Seidel C., Heider S., Hau P., Glasow A., Dietzsch S., Kortmann R.-D. (2021). Radiotherapy in medulloblastoma—Evolution of treatment, current concepts and future perspectives. Cancers.

[48] Mani S., Chatterjee A., Dasgupta A., Shirsat N., Pawar A., Epari S., Sahay A., Sahu A., Moiyadi A., Prasad M. (2024). Clinico-RADIOLOGICAL OUTCOMes in WNT-subgroup medulloblastoma. Diagnostics.

[49] Gwynne W.D., Suk Y., Custers S., Mikolajewicz N., Chan J.K., Zador Z., Chafe S.C., Zhai K., Escudero L., Zhang C. (2022). Cancer-selective metabolic vulnerabilities in MYC-amplified medulloblastoma. Cancer Cell.

[50] Yan H., Zabih V., Bartels U., Das S., Nathan P., Gupta S. (2022). Prognostic factors related to overall survival in adolescent and young adults with medulloblastoma: A systematic review. Neuro-Oncol. Adv..

[51] Bevacqua E., Farshchi J., Niklison-Chirou M.V., Tucci P. (2021). Role of MicroRNAs in the development and progression of the four medulloblastoma subgroups. Cancers.

[52] Panwalkar P., Moiyadi A., Goel A., Shetty P., Goel N., Sridhar E., Shirsat N. (2015). MiR-206, a cerebellum enriched miRNA is downregulated in all medulloblastoma subgroups and its overexpression is necessary for growth inhibition of medulloblastoma cells. J. Mol. Neurosci..

[53] Weeraratne S.D., Amani V., Teider N., Pierre-Francois J., Winter D., Kye M.J., Sengupta S., Archer T., Remke M., Bai A.H. (2012). Pleiotropic effects of miR-183~ 96~ 182 converge to regulate cell survival, proliferation and migration in medulloblastoma. Acta Neuropathol..

[54] Xu H., Zhao G., Zhang Y., Jiang H., Wang W., Zhao D., Hong J., Yu H., Qi L. (2019). Mesenchymal stem cell-derived exosomal microRNA-133b suppresses glioma progression via Wnt/β-catenin signaling pathway by targeting EZH2. Stem Cell Res. Ther..

[55] Yogi K., Sridhar E., Goel N., Jalali R., Goel A., Moiyadi A., Thorat R., Panwalkar P., Khire A., Dasgupta A. (2015). MiR-148a, a microRNA upregulated in the WNT subgroup tumors, inhibits invasion and tumorigenic potential of medulloblastoma cells by targeting Neuropilin 1. Oncoscience.

[56] Besharat Z.M., Sabato C., Po A., Gianno F., Abballe L., Napolitano M., Miele E., Giangaspero F., Vacca A., Catanzaro G. (2018). Low expression of miR-466f-3p sustains epithelial to mesenchymal transition in sonic hedgehog medulloblastoma stem cells through Vegfa-Nrp2 signaling pathway. Front. Pharmacol..

[57] Westphal M.S., Lee E., Schadt E.E., Sholler G.S., Zhu J. (2021). Identification of Let-7 miRNA activity as a prognostic biomarker of SHH medulloblastoma. Cancers.

[58] Murphy B.L., Obad S., Bihannic L., Ayrault O., Zindy F., Kauppinen S., Roussel M.F. (2013). Silencing of the miR-17∼92 cluster family inhibits medulloblastoma progression. Cancer Res..

[59] Northcott P.A., Robinson G.W., Kratz C.P., Mabbott D.J., Pomeroy S.L., Clifford S.C., Rutkowski S., Ellison D.W., Malkin D., Taylor M.D. (2019). Medulloblastoma. Nat. Rev. Dis. Primers.

[60] Laneve P., Caffarelli E. (2020). The non-coding side of medulloblastoma. Front. Cell Dev. Biol..

[61] Zhang Z., Li S., Cheng S.Y. (2013). The miR-183∼96∼182 cluster promotes tumorigenesis in a mouse model of medulloblastoma. J. Biomed. Res..

[62] Paul R., Bharambe H., Shirsat N.V. (2020). Autophagy inhibition impairs the invasion potential of medulloblastoma cells. Mol. Biol. Rep..

[63] Singh S.V., Dakhole A.N., Deogharkar A., Kazi S., Kshirsagar R., Goel A., Moiyadi A., Jalali R., Sridhar E., Gupta T. (2017). Restoration of miR-30a expression inhibits growth, tumorigenicity of medulloblastoma cells accompanied by autophagy inhibition. Biochem. Biophys. Res. Commun..

[64] Aneja K.K. (2024). Mining the epigenetic landscape of medulloblastoma. Int. J. Epigenet..

[65] Senfter D., Samadaei M., Mader R.M., Gojo J., Peyrl A., Krupitza G., Kool M., Sill M., Haberler C., Ricken G. (2019). High impact of miRNA-4521 on FOXM1 expression in medulloblastoma. Cell Death Dis..

[66] Gershanov S., Toledano H., Michowiz S., Barinfeld O., Pinhasov A., Goldenberg-Cohen N., Salmon-Divon M. (2018). MicroRNA–mRNA expression profiles associated with medulloblastoma subgroup 4. Cancer Manag. Res..

[67] Beylerli O., Musaev E., Ilyasova T., Sufianov A. (2025). lncRNAs and circRNAs: Emerging Players in Pediatric Medulloblastoma Pathology. Curr. Med. Chem..

[68] Nejadi Orang F., Abdoli Shadbad M. (2024). CircRNA and lncRNA-associated competing endogenous RNA networks in medulloblastoma: A scoping review. Cancer Cell Int..

[69] Li B., Shen M., Yao H., Chen X., Xiao Z. (2019). Long noncoding RNA TP73-AS1 modulates medulloblastoma progression in vitro and in vivo by sponging miR-494-3p and targeting EIF5A2. OncoTargets Ther..

[70] Varon M., Levy T., Mazor G., Ben David H., Marciano R., Krelin Y., Prasad M., Elkabets M., Pauck D., Ahmadov U. (2019). The long noncoding RNA TP73-AS1 promotes tumorigenicity of medulloblastoma cells. Int. J. Cancer.

[71] Zhang J., Li N., Fu J., Zhou W. (2020). Long noncoding RNA HOTAIR promotes medulloblastoma growth, migration and invasion by sponging miR-1/miR-206 and targeting YY1. Biomed. Pharmacother..

[72] Bartl J., Zanini M., Bernardi F., Forget A., Blümel L., Talbot J., Picard D., Qin N., Cancila G., Gao Q. (2022). The HHIP-AS1 lncRNA promotes tumorigenicity through stabilization of dynein complex 1 in human SHH-driven tumors. Nat. Commun..

[73] Do A.D., Wu K.-S., Chu S.-S., Giang L.H., Lin Y.-L., Chang C.-C., Wong T.-T., Hsieh C.-L., Sung S.-Y. (2024). LOXL1-AS1 contributes to metastasis in sonic-hedgehog medulloblastoma by promoting cancer stem-like phenotypes. J. Exp. Clin. Cancer Res..

[74] Gao R., Zhang R., Zhang C., Zhao L., Zhang Y. (2018). Long noncoding RNA CCAT1 promotes cell proliferation and metastasis in human medulloblastoma via MAPK pathway. Tumori J..

[75] Ghafouri-Fard S., Safarzadeh A., Hussen B.M., Taheri M., Mokhtari M. (2023). Contribution of CRNDE lncRNA in the development of cancer and the underlying mechanisms. Pathol.-Res. Pract..

[76] Hosseini N.F., Manoochehri H., Khoei S.G., Sheykhhasan M. (2021). The functional role of long non-coding RNA UCA1 in human multiple cancers: A review study. Curr. Mol. Med..

[77] Laneve P., Po A., Favia A., Legnini I., Alfano V., Rea J., Di Carlo V., Bevilacqua V., Miele E., Mastronuzzi A. (2017). The long noncoding RNA linc-NeD125 controls the expression of medulloblastoma driver genes by microRNA sponge activity. Oncotarget.

[78] Lee B., Katsushima K., Pokhrel R., Yuan M., Stapleton S., Jallo G., Wechsler-Reya R.J., Eberhart C.G., Ray A., Perera R.J. (2022). The long non-coding RNA SPRIGHTLY and its binding partner PTBP1 regulate exon 5 skipping of SMYD3 transcripts in group 4 medulloblastomas. Neuro-Oncol. Adv..

[79] Mutlu M., Tekin C., Ak Aksoy S., Taskapilioglu M.O., Kaya S., Balcin R.N., Ocak P.E., Kocaeli H., Bekar A., Tolunay S. (2022). Long non-coding RNAs as a predictive markers of group 3 medulloblastomas. Neurol. Res..

[80] Shi P.-F., Ji H.-L., Luo Y.-K., Mao T.-M., Chen X., Zhou K.-Y. (2017). Effect of long noncoding RNA SPRY4-IT1 on proliferation and metastasis of medulloblastoma. Zhongguo Ying Yong Sheng Li Xue Za Zhi = Zhongguo Yingyong Shenglixue Zazhi = Chin. J. Appl. Physiol..

[81] Ge J., Wang B., Zhao S., Xu J. (2022). Inhibition of lncRNA NEAT1 sensitizes medulloblastoma cells to cisplatin through modulating the miR-23a-3p-glutaminase (GLS) axis. Bioengineered.

[82] Zhang Y., Wang T., Wang S., Xiong Y., Zhang R., Zhang X., Zhao J., Yang A.-G., Wang L., Jia L. (2018). Nkx2-2as suppression contributes to the pathogenesis of sonic hedgehog medulloblastoma. Cancer Res..

[83] Liu X.-c., Wang F.-C., Wang J.-H., Zhao J.-Y., Ye S.-Y. (2022). The circular RNA circSKA3 facilitates the malignant biological behaviors of medulloblastoma via miR-520 h/CDK6 pathway. Mol. Biotechnol..

[84] Wang X., Xu D., Pei X., Zhang Y., Zhang Y., Gu Y., Li Y. (2020). CircSKA3 modulates FOXM1 to facilitate cell proliferation, migration, and invasion while confine apoptosis in medulloblastoma via miR-383-5p. Cancer Manag. Res..

[85] Katsushima K., Pokhrel R., Mahmud I., Yuan M., Murad R., Baral P., Zhou R., Chapagain P., Garrett T., Stapleton S. (2023). The oncogenic circular RNA circ_63706 is a potential therapeutic target in sonic hedgehog-subtype childhood medulloblastomas. Acta Neuropathol. Commun..

[86] Pokhrel R., Katsushima K., Stapleton S., Jallo G., Raabe E., Eberhart C.G., Perera R.J. (2022). MEDB-02. The identification and functional characterization of circular RNA Circ_63706 in sonic hedgehog medulloblastomas. Neuro-Oncol..

[87] Yin H., Zhao Y., Han X., Li Q., Dong Q., Liu Y., Wang X., Yuan G., Pan Y. (2023). Circ_103128 is associated with the tumorigenesis of medulloblastoma. J. Cancer Res. Clin. Oncol..

[88] Lv T., Miao Y.F., Jin K., Han S., Xu T.Q., Qiu Z.L., Zhang X.H. (2018). Dysregulated circular RNAs in medulloblastoma regulate proliferation and growth of tumor cells via host genes. Cancer Med..

[89] Feng Y., Feng X., Lv Y. (2024). Worldwide Burden of Retinoblastoma from 1990 to 2021. Ophthalmic Res..

[90] Li N., Wang Y.-Z., Zhang Y., Zhang W.-L., Huang D.-S. (2024). Characteristics of patients with recurrent retinoblastoma: A survival analysis. BMC Cancer.

[91] Zhang S., Huang G., Li X., Zhang Z., Peng K., Zhu L., Zhang C., Niu T.-t. (2025). Global, regional and national retinoblastoma burden in children under 10 years of age from 1990 to 2021: Trend analysis based on the Global Burden of Disease Study 2021. PLoS ONE.

[92] Fabian I.D., Abdallah E., Abdullahi S.U., Abdulqader R.A., Abdulrahaman A.A., Abouelnaga S., Ademola-Popoola D.S., Adio A., Afifi M.A., Afshar A.R. (2022). The Global Retinoblastoma Outcome Study: A prospective, cluster-based analysis of 4064 patients from 149 countries. Lancet Glob. Health.

[93] Wang L., Chen J., Shen Y., Hooi G.L.M., Wu S., Xu F., Pei H., Sheng J., Zhu T., Ye J. (2025). Incidence, mortality, and global burden of retinoblastoma in 204 countries worldwide from 1990 to 2021: Data and systematic analysis from the Global Burden of Disease Study 2021. Neoplasia.

[94] Marković L., Bukovac A., Varošanec A.M., Šlaus N., Pećina-Šlaus N. (2023). Genetics in ophthalmology: Molecular blueprints of retinoblastoma. Hum. Genom..

[95] Pallavi R., Soni B.L., Jha G.K., Sanyal S., Fatima A., Kaliki S. (2025). Tumor heterogeneity in retinoblastoma: A literature review. Cancer Metastasis Rev..

[96] Faranoush M., Naseripour M., Faranoush P., Davoodi-Moghaddam Z., Jahandideh A., Sadighnia N., Daneshjou D., Shams P., Sedaghat A., Mirshahi R. (2025). Delving Into Retinoblastoma Genetics: Discovery of Novel Mutations and Their Clinical Impact: Retrospective Cohort Study. Cancer Med..

[97] Guo X., Wang L., Beeraka N.M., Liu C., Zhao X., Zhou R., Yu H., Fan R., Liu J. (2024). Incidence trends, clinicopathologic characteristics, and overall survival prediction in retinoblastoma children: SEER prognostic nomogram analysis. Oncologist.

[98] Mohammad M., Mehyar M., Halalsheh H., Shehada R., Al Adawi O., Khzouz J., Jaradat I., Al-Hussaini M., Sultan I., Alnawaiseh I. (2024). The Impact of Tumor Laterality (Unilateral vs. Bilateral) on Presentation and Management Outcome in Patients with Retinoblastoma. J. Clin. Med..

[99] Chandra K., Raval V., Reddy P V.A., Kaliki S. (2022). Primary macular retinoblastoma: Clinical presentation and treatment outcomes. J. Vitreoretin. Dis..

[100] Cruz-Gálvez C.C., Ordaz-Favila J.C., Villar-Calvo V.M., Cancino-Marentes M.E., Bosch-Canto V. (2022). Retinoblastoma: Review and new insights. Front. Oncol..

[101] Gu H., Wang Y., Huang D., Ji X., Zhang Y., Ma J., Li M., Zhang W., Hu H., Li J. (2023). Clinical characteristics and image manifestations of a rare retinoblastoma with a bone metastasis. Cancer Manag. Res..

[102] Kakarala C.L., Raval V.R., Mallu A., Rao R., Gavara S., Reddy V.A.P., Mishra D.K., Jakati S., Kaliki S. (2023). Metastatic retinoblastoma at presentation: Clinical presentation, treatment, and outcomes. Oman J. Ophthalmol..

[103] Kritfuangfoo T., Rojanaporn D. (2024). Update on chemotherapy modalities for retinoblastoma: Progress and challenges. Asia-Pac. J. Ophthalmol..

[104] Zhou M., Tang J., Fan J., Wen X., Shen J., Jia R., Chai P., Fan X. (2024). Recent progress in retinoblastoma: Pathogenesis, presentation, diagnosis and management. Asia-Pac. J. Ophthalmol..

[105] He X., Han M., Zhou M., Chai P., Guo L., Fan J., Wen X., Fan X. (2025). Effect of intra-arterial chemotherapy drug regimens on globe salvage outcomes of retinoblastoma patients. Br. J. Ophthalmol..

[106] Teixeira L.F., Macedo C.R., Fonseca J.R., Morales B., Mangeon M.K., Miranda B.A., Casaroli-Marano R., Sallum J.M. (2025). Intraarterial Chemotherapy for Retinoblastoma, Outcomes Analysis in 357 Eyes: Thirteen Years of Experience in a Referral Center in Brazil. Ophthalmol. Retin..

[107] Fabius A.W., van Hoefen Wijsard M., van Leeuwen F.E., Moll A.C. (2021). Subsequent malignant neoplasms in retinoblastoma survivors. Cancers.

[108] Sun J., Gu X., Wang L. (2024). Incidence of second primary cancers in patients with retinoblastoma: A systematic review and meta-analysis. Front. Oncol..

[109] Kong L., Sun Y., Chen M., Dai Y., Liu Z. (2020). Downregulation of microRNA-320a inhibits proliferation and induces apoptosis of retinoblastoma cells via targeting TUSC3. Exp. Ther. Med..

[110] Gao Y., Du P. (2024). miR-889-3p targeting BMPR2 promotes the development of retinoblastoma via JNK/MAPK/ERK signaling. Sci. Rep..

[111] Wan W., Wan W., Long Y., Li Q., Jin X., Wan G., Zhang F., Lv Y., Zheng G., Li Z. (2019). MiR-25-3p promotes malignant phenotypes of retinoblastoma by regulating PTEN/Akt pathway. Biomed. Pharmacother..

[112] Liu S., Wen C. (2022). miR-141-3p promotes retinoblastoma progression via inhibiting sushi domain-containing protein 2. Bioengineered.

[113] Li C., Zhao J., Sun W. (2020). microRNA-222-mediated VHL downregulation facilitates retinoblastoma chemoresistance by increasing HIF1α expression. Investig. Ophthalmol. Vis. Sci..

[114] Song L., Huang Y., Zhang X., Han S., Hou M., Li H. (2020). Downregulation of microRNA-224-3p hampers retinoblastoma progression via activation of the hippo-YAP signaling pathway by increasing LATS2. Investig. Ophthalmol. Vis. Sci..

[115] Sun Z., Zhang A., Zhang L. (2019). Inhibition of microRNA-492 attenuates cell proliferation and invasion in retinoblastoma via directly targeting LATS2. Mol. Med. Rep..

[116] Chen S., Chen X., Luo Q., Liu X., Wang X., Cui Z., He A., He S., Jiang Z., Wu N. (2021). Retinoblastoma cell-derived exosomes promote angiogenesis of human vesicle endothelial cells through microRNA-92a-3p. Cell Death Dis..

[117] Yin W., Gao F., Zhang S. (2020). MicroRNA-34a inhibits the proliferation and promotes the chemosensitivity of retinoblastoma cells by downregulating Notch1 expression. Mol. Med. Rep..

[118] Zhang S., Cui Z. (2021). MicroRNA-34b-5p inhibits proliferation, stemness, migration and invasion of retinoblastoma cells via Notch signaling. Exp. Ther. Med..

[119] Guo L., Bai Y., Ni T., Li Y., Cao R., Ji S., Li S. (2021). MicroRNA-153-3p suppresses retinoblastoma cell growth and invasion via targeting the IGF1R/Raf/MEK and IGF1R/PI3K/AKT signaling pathways. Int. J. Oncol..

[120] Zhao D., Cui Z. (2019). MicroRNA-361-3p regulates retinoblastoma cell proliferation and stemness by targeting hedgehog signaling. Exp. Ther. Med..

[121] Hao F., Mou Y., Zhang L., Wang S., Yang Y. (2018). LncRNA AFAP1-AS1 is a prognostic biomarker and serves as oncogenic role in retinoblastoma. Biosci. Rep..

[122] Su S., Gao J., Wang T., Wang J., Li H., Wang Z. (2015). Long non-coding RNA BANCR regulates growth and metastasis and is associated with poor prognosis in retinoblastoma. Tumor Biol..

[123] Lin X., Huang X., Wang L., Liu W. (2022). The long noncoding RNA MALAT1/microRNA-598-3p axis regulates the proliferation and apoptosis of retinoblastoma cells through the PI3K/AKT pathway. Mol. Vis..

[124] Wang L., Zhang Y., Xin X. (2020). Long non-coding RNA MALAT1 aggravates human retinoblastoma by sponging miR-20b-5p to upregulate STAT3. Pathol.-Res. Pract..

[125] Liu Y., Xin Z., Zhang K., Jin X., Wang D. (2024). LncRNA NEAT1 promotes angiogenesis of retinoblastoma cells through regulation of the miR-106a/HIF-1α axis. Heliyon.

[126] Luan L., Hu Q., Wang Y., Lu L., Ling J. (2021). Knockdown of lncRNA NEAT1 expression inhibits cell migration, invasion and EMT by regulating the miR-24-3p/LRG1 axis in retinoblastoma cells. Exp. Ther. Med..

[127] Wang Y., Sun D., Sheng Y., Guo H., Meng F., Song T. (2020). XIST promotes cell proliferation and invasion by regulating miR-140-5p and SOX4 in retinoblastoma. World J. Surg. Oncol..

[128] Xu Y., Fu Z., Gao X., Wang R., Li Q. (2021). Long non-coding RNA XIST promotes retinoblastoma cell proliferation, migration, and invasion by modulating microRNA-191-5p/brain derived neurotrophic factor. Bioengineered.

[129] Peng X., Yan J., Cheng F. (2020). LncRNA TMPO-AS1 up-regulates the expression of HIF-1α and promotes the malignant phenotypes of retinoblastoma cells via sponging miR-199a-5p. Pathol.-Res. Pract..

[130] Wang Z., Liang X., Yi G., Wu T., Sun Y., Zhang Z., Fu M. (2024). Bioinformatics analysis proposes a possible role for long noncoding RNA MIR17HG in retinoblastoma. Cancer Rep..

[131] Yin X., Liao Y., Xiong W., Zhang Y., Zhou Y., Yang Y. (2020). Hypoxia-induced lncRNA ANRIL promotes cisplatin resistance in retinoblastoma cells through regulating ABCG2 expression. Clin. Exp. Pharmacol. Physiol..

[132] Fu K., Zhang K., Zhang X. (2022). LncRNA HOTAIR facilitates proliferation and represses apoptosis of retinoblastoma cells through the miR-20b-5p/RRM2/PI3K/AKT axis. Orphanet J. Rare Dis..

[133] Song J., Zhang Z. (2021). Long non-coding RNA SNHG20 promotes cell proliferation, migration and invasion in retinoblastoma via the miR-335-5p/E2F3 axis. Mol. Med. Rep..

[134] Wang B., Cai R., Sun T., Yang Z., Zhang H. (2024). Long non-coding RNA MIMT1 promotes retinoblastoma proliferation via sponging miR-153-5p to upregulate FGF2. Heliyon.

[135] Wu X.-Z., Cui H.-P., Lv H.-J., Feng L. (2019). Knockdown of lncRNA PVT1 inhibits retinoblastoma progression by sponging miR-488-3p. Biomed. Pharmacother..

[136] Yan G., Su Y., Ma Z., Yu L., Chen N. (2019). Long noncoding RNA LINC00202 promotes tumor progression by sponging miR-3619-5p in retinoblastoma. Cell Struct. Funct..

[137] Bi L.-L., Han F., Zhang X.-M., Li Y.-Y. (2018). LncRNA MT1JP acts as a tumor inhibitor via reciprocally regulating Wnt/β-Catenin pathway in retinoblastoma. Eur. Rev. Med. Pharmacol. Sci..

[138] Gao Y., Chen X., Zhang J. (2022). LncRNA MEG3 inhibits retinoblastoma invasion and metastasis by inducing β-catenin degradation. Am. J. Cancer Res..

[139] Yan X., Jia H., Zhao J. (2023). LncRNA MEG3 attenuates the malignancy of retinoblastoma cells through inactivating PI3K/Akt/mTOR signaling pathway. Exp. Eye Res..

[140] Gao Y.-X., Gao H.-X., Xu X.-Y., Ding F.-K. (2020). Effects of lncRNA MALAT1 and lncRNA NKILA on proliferation, invasion and apoptosis of retinoblastoma. Eur. Rev. Med. Pharmacol. Sci..

[141] Lyu X., Ma Y., Wu F., Wang L., Wang L. (2019). LncRNA NKILA inhibits retinoblastoma by downregulating lncRNA XIST. Curr. Eye Res..

[142] Jiang Y., Xiao F., Wang L., Wang T., Chen L. (2021). Circular RNA has_circ_0000034 accelerates retinoblastoma advancement through the miR-361-3p/ADAM19 axis. Mol. Cell. Biochem..

[143] Wang H., Li M., Cui H., Song X., Sha Q. (2020). CircDHDDS/miR-361-3p/WNT3A Axis promotes the development of retinoblastoma by regulating proliferation, cell cycle, migration, and invasion of retinoblastoma cells. Neurochem. Res..

[144] Liang T., Fan M., Meng Z., Sun B., Mi S., Gao X. (2022). Circ_0000527 drives retinoblastoma progression by regulating miR-1236-3p/SMAD2 pathway. Curr. Eye Res..

[145] Zhang L., Wu J., Li Y., Jiang Y., Wang L., Chen Y., Lv Y., Zou Y., Ding X. (2020). Circ_0000527 promotes the progression of retinoblastoma by regulating miR-646/LRP6 axis. Cancer Cell Int..

[146] Han Q., Ma L., Shao L., Wang H., Feng M. (2022). Circ_0075804 regulates the expression of LASP1 by targeting miR-1287-5p and thus affects the biological process of retinoblastoma. Curr. Eye Res..

[147] Huang Y., Xue B., Pan J., Shen N. (2021). Circ-E2F3 acts as a ceRNA for miR-204-5p to promote proliferation, metastasis and apoptosis inhibition in retinoblastoma by regulating ROCK1 expression. Exp. Mol. Pathol..

[148] An D., Yang J., Ma L. (2022). circRNF20 aggravates the malignancy of retinoblastoma depending on the regulation of miR-132-3p/PAX6 axis. Open Med..

[149] Fu C., Wang S., Jin L., Zhang M., Li M. (2021). CircTET1 inhibits retinoblastoma progression via targeting miR-492 and miR-494-3p through Wnt/β-catenin signaling pathway. Curr. Eye Res..

[150] Xing L., Zhang L., Feng Y., Cui Z., Ding L. (2018). Downregulation of circular RNA hsa_circ_0001649 indicates poor prognosis for retinoblastoma and regulates cell proliferation and apoptosis via AKT/mTOR signaling pathway. Biomed. Pharmacother..

[151] Xu L., Long H., Zhou B., Jiang H., Cai M. (2021). CircMKLN1 suppresses the progression of human retinoblastoma by modulation of miR-425-5p/PDCD4 axis. Curr. Eye Res..

[152] Zhang H., Qiu X., Song Z., Lan L., Ren X., Ye B. (2022). CircCUL2 suppresses retinoblastoma cells by regulating miR-214-5p/E2F2 Axis. Anti-Cancer Drugs.

[153] Parham D.M., Barr F.G. (2013). Classification of rhabdomyosarcoma and its molecular basis. Adv. Anat. Pathol..

[154] Dehner C.A., Rudzinski E.R., Davis J.L. (2024). Rhabdomyosarcoma: Updates on classification and the necessity of molecular testing beyond immunohistochemistry. Hum. Pathol..

[155] Skapek S.X., Ferrari A., Gupta A.A., Lupo P.J., Butler E., Shipley J., Barr F.G., Hawkins D.S. (2019). Rhabdomyosarcoma. Nat. Rev. Dis. Primers.

[156] Rudzinski E.R., Anderson J.R., Hawkins D.S., Skapek S.X., Parham D.M., Teot L.A. (2015). The World Health Organization classification of skeletal muscle tumors in pediatric rhabdomyosarcoma: A report from the Children’s Oncology Group. Arch. Pathol. Lab. Med..

[157] Raze T., Lapouble E., Lacour B., Guissou S., Defachelles A.S., Gaspar N., Delattre O., Pierron G., Desandes E. (2023). PAX–FOXO1 fusion status in children and adolescents with alveolar rhabdomyosarcoma: Impact on clinical, pathological, and survival features. Pediatr. Blood Cancer.

[158] Zarrabi A., Perrin D., Kavoosi M., Sommer M., Sezen S., Mehrbod P., Bhushan B., Machaj F., Rosik J., Kawalec P. (2023). Rhabdomyosarcoma: Current therapy, challenges, and future approaches to treatment strategies. Cancers.

[159] Haduong J.H., Heske C.M., Allen-Rhoades W., Xue W., Teot L.A., Rodeberg D.A., Donaldson S.S., Weiss A., Hawkins D.S., Venkatramani R. (2022). An update on rhabdomyosarcoma risk stratification and the rationale for current and future Children’s Oncology Group clinical trials. Pediatr. Blood Cancer.

[160] Sumegi J., Streblow R., Frayer R.W., Cin P.D., Rosenberg A., Meloni-Ehrig A., Bridge J.A. (2010). Recurrent t(2;2) and t(2;8) translocations in rhabdomyosarcoma without the canonical *PAX-FOXO1* fuse *PAX3* to members of the nuclear receptor transcriptional coactivator family. Genes Chromosomes Cancer.

[161] Agaram N.P., Huang S.C., Tap W.D., Wexler L.H., Antonescu C.R. (2022). Clinicopathologic and survival correlates of embryonal rhabdomyosarcoma driven by RAS/RAF mutations. Genes Chromosomes Cancer.

[162] Yu L., He L., Zhang N. (2025). BCOR Mutations Identify a Clinically Aggressive Subset of Pediatric Rhabdomyosarcoma. Fetal Pediatr. Pathol..

[163] Kaseb H., Kuhn J., Gasalberti D.P., Babiker H.M. (2024). Rhabdomyosarcoma. StatPearls.

[164] Ognjanovic S., Olivier M., Bergemann T.L., Hainaut P. (2012). Sarcomas in TP53 germline mutation carriers: A review of the IARC TP53 database. Cancer.

[165] Plon S., Malkin D. (2006). Childhood cancer and heredity. Principles and Practice of Pediatric Oncology.

[166] Cao L., Yu Y., Bilke S., Walker R.L., Mayeenuddin L.H., Azorsa D.O., Yang F., Pineda M., Helman L.J., Meltzer P.S. (2010). Genome-wide identification of *PAX3*-FKHR binding sites in rhabdomyosarcoma reveals candidate target genes important for development and cancer. Cancer Res..

[167] Hanna J.A., Garcia M.R., Lardennois A., Leavey P.J., Maglic D., Fagnan A., Go J.C., Roach J., Wang Y.-D., Finkelstein D. (2018). PAX3-FOXO1 drives miR-486-5p and represses miR-221 contributing to pathogenesis of alveolar rhabdomyosarcoma. Oncogene.

[168] Ramadan F., Saab R., Hussein N., Clézardin P., Cohen P.A., Ghayad S.E. (2022). Non-coding RNA in rhabdomyosarcoma progression and metastasis. Front. Oncol..

[169] Bharathy N., Berlow N.E., Wang E., Abraham J., Settelmeyer T.P., Hooper J.E., Svalina M.N., Ishikawa Y., Zientek K., Bajwa Z. (2018). The HDAC3–SMARCA4–miR-27a axis promotes expression of the *PAX3*: *FOXO1* fusion oncogene in rhabdomyosarcoma. Sci. Signal..

[170] Hanna J., Garcia M., Go J., Finkelstein D., Kodali K., Pagala V., Wang X., Peng J., Hatley M. (2016). PAX7 is a required target for microRNA-206-induced differentiation of fusion-negative rhabdomyosarcoma. Cell Death Dis..

[171] Di Paolo V., Paolini A., Galardi A., Gasparini P., De Cecco L., Colletti M., Lampis S., Raieli S., De Stefanis C., Miele E. (2024). Plasma-derived extracellular vesicles miR-335–5p as potential diagnostic biomarkers for fusion-positive rhabdomyosarcoma. J. Exp. Clin. Cancer Res..

[172] Pan Y., Li J., Lou S., Chen W., Lin Y., Shen N., Li Y. (2022). Down-regulated miR-130a/b attenuates rhabdomyosarcoma proliferation via PPARG. Front. Mol. Biosci..

[173] Pozzo E., Giarratana N., Sassi G., Elmastas M., Killian T., Wang C.-c., Marini V., Ronzoni F., Yustein J., Uyttebroeck A. (2021). Upregulation of miR181a/miR212 improves myogenic commitment in murine fusion-negative rhabdomyosarcoma. Front. Physiol..

[174] Gasparini P., Fortunato O., De Cecco L., Casanova M., Iannó M.F., Carenzo A., Centonze G., Milione M., Collini P., Boeri M. (2019). Age-related alterations in immune contexture are associated with aggressiveness in rhabdomyosarcoma. Cancers.

[175] Molist C., Navarro N., Giralt I., Zarzosa P., Gallo-Oller G., Pons G., Magdaleno A., Moreno L., Guillén G., Hladun R. (2020). miRNA-7 and miRNA-324-5p regulate alpha9-Integrin expression and exert anti-oncogenic effects in rhabdomyosarcoma. Cancer Lett..

[176] Skrzypek K., Nieszporek A., Badyra B., Lasota M., Majka M. (2021). Enhancement of myogenic differentiation and inhibition of rhabdomyosarcoma progression by miR-28-3p and miR-193a-5p regulated by SNAIL. Mol. Ther. Nucleic Acids.

[177] Casanova M., Pontis F., Ghidotti P., Petraroia I., Venturini L.V., Bergamaschi L., Chiaravalli S., De Cecco L., Massimino M., Sozzi G. (2022). MiR-223 exclusively impairs in vitro tumor growth through IGF1R modulation in rhabdomyosarcoma of adolescents and young adults. Int. J. Mol. Sci..

[178] Wang Y., Zhang L., Pang Y., Song L., Shang H., Li Z., Liu Q., Zhang Y., Wang X., Li Q. (2020). MicroRNA-29 family inhibits rhabdomyosarcoma formation and progression by regulating GEFT function. Am. J. Transl. Res..

[179] Tombolan L., Millino C., Pacchioni B., Cattelan M., Zin A., Bonvini P., Bisogno G. (2020). Circulating miR-26a as potential prognostic biomarkers in pediatric rhabdomyosarcoma. Front. Genet..

[180] O’Brien E.M., Selfe J.L., Martins A.S., Walters Z.S., Shipley J.M. (2018). The long non-coding RNA MYCNOS-01 regulates MYCN protein levels and affects growth of MYCN-amplified rhabdomyosarcoma and neuroblastoma cells. BMC Cancer.

[181] Dey B.K., Pfeifer K., Dutta A. (2014). The H19 long noncoding RNA gives rise to microRNAs miR-675-3p and miR-675-5p to promote skeletal muscle differentiation and regeneration. Genes Dev..

[182] Tarnowski M., Tkacz M., Czerewaty M., Poniewierska-Baran A., Grymuła K., Ratajczak M.Z. (2015). 5-Azacytidine inhibits human rhabdomyosarcoma cell growth by downregulating insulin-like growth factor 2 expression and reactivating the H19 gene product miR-675, which negatively affects insulin-like growth factors and insulin signaling. Int. J. Oncol..

[183] Jin J.J., Lv W., Xia P., Xu Z.Y., Zheng A.D., Wang X.J., Wang S.S., Zeng R., Luo H.M., Li G.L. (2018). Long noncoding RNA SYISL regulates myogenesis by interacting with polycomb repressive complex 2. Proc. Natl. Acad. Sci. USA.

[184] Wang S., Zuo H., Jin J., Lv W., Xu Z., Fan Y., Zhang J., Zuo B. (2019). Long noncoding RNA Neat1 modulates myogenesis by recruiting Ezh2. Cell Death Dis..

[185] Steiner A.J., Zheng Y., Tang Y. (2023). Characterization of a rhabdomyosarcoma reveals a critical role for SMG7 in cancer cell viability and tumor growth. Sci. Rep..

[186] Yoon J.-H., Abdelmohsen K., Gorospe M. (2014). Functional interactions among microRNAs and long noncoding RNAs. Semin. Cell Dev. Biol..

[187] Centrón-Broco A., Rossi F., Grelloni C., Garraffo R., Dattilo D., Giuliani A., Di Timoteo G., Colantoni A., Bozzoni I., Beltran Nebot M. (2023). CircAFF1 is a circular RNA with a role in alveolar rhabdomyosarcoma cell migration. Biomedicines.

[188] Rossi F., Centrón-Broco A., Dattilo D., Di Timoteo G., Guarnacci M., Colantoni A., Beltran Nebot M., Bozzoni I. (2021). CircVAMP3: A circRNA with a role in alveolar rhabdomyosarcoma cell cycle progression. Genes.

[189] Rossi F., Legnini I., Megiorni F., Colantoni A., Santini T., Morlando M., Di Timoteo G., Dattilo D., Dominici C., Bozzoni I. (2019). Circ-ZNF609 regulates G1-S progression in rhabdomyosarcoma. Oncogene.

[190] Pio L., O’Neill A.F., Woodley H., Murphy A.J., Tiao G., Franchi-Abella S., Fresneau B., Watanabe K., Alaggio R., Lopez-Terrada D. (2025). Hepatoblastoma. Nat. Rev. Dis. Primers.

[191] Klein S.D., DeMarchis M., Linn R.L., MacFarland S.P., Kalish J.M. (2023). Occurrence of hepatoblastomas in patients with Beckwith–Wiedemann Spectrum (BWSp). Cancers.

[192] Zhu L.-r., Zheng W., Gao Q., Chen T., Pan Z.-b., Cui W., Cai M., Fang H. (2022). Epigenetics and genetics of hepatoblastoma: Linkage and treatment. Front. Genet..

[193] Yang L., Yu Z.-Q., Zhang Y.-Z., Deng Y.-Q., Chen X., Liu H.-Y., Bai X.-Y., Zhao H. (2025). Global, regional, and national burden of hepatoblastoma, 1990–2021: A systematic analysis of the global burden of disease study 2021. Int. J. Surg..

[194] Hellmann Z.J., Rehman S., Brown L.M., Vasquez J.C., Solomon D.G., Christison-Lagay E.R. (2025). Relationship Between Total Parenteral Nutrition, Ventilation, and Hepatoblastoma: A Study of 258,929 Neonatal Intensive Care Unit Admissions. Pediatr. Blood Cancer.

[195] Fan L., Na J., Shi T., Liao Y. (2025). Hepatoblastoma: From molecular mechanisms to therapeutic strategies. Curr. Oncol..

[196] Nagae G., Yamamoto S., Fujita M., Fujita T., Nonaka A., Umeda T., Fukuda S., Tatsuno K., Maejima K., Hayashi A. (2021). Genetic and epigenetic basis of hepatoblastoma diversity. Nat. Commun..

[197] Morgan Auld F., Sergi C.M. (2022). Surgical pathology diagnostic pitfalls of hepatoblastoma. Int. J. Surg. Pathol..

[198] Demir S., Hotes A., Schmid T., Cairo S., Indersie E., Pisano C., Hiyama E., Hishiki T., Vokuhl C., Branchereau S. (2024). Drug prioritization identifies panobinostat as a tailored treatment element for patients with metastatic hepatoblastoma. J. Exp. Clin. Cancer Res..

[199] Srinivasan S., Prasad M., Parambil B.C., Shrimal A., Gollamudi V.R.M., Subramani V., Ramadwar M., Khanna N., Baheti A.D., Gala K. (2023). Treatment outcomes and prognostic factors in children with hepatoblastoma using a risk-stratified approach. Pediatr. Blood Cancer.

[200] Kahla J.A., Siegel D.A., Dai S., Lupo P.J., Foster J.H., Scheurer M.E., Heczey A.A. (2022). Incidence and 5-year survival of children and adolescents with hepatoblastoma in the United States. Pediatr. Blood Cancer.

[201] Aghajanzadeh T., Tebbi K., Talkhabi M. (2021). Identification of potential key genes and miRNAs involved in Hepatoblastoma pathogenesis and prognosis. J. Cell Commun. Signal..

[202] Feng S.G., Bhandari R., Ya L., Zhixuan B., Qiuhui P., Jiabei Z., Sewi M., Ni Z., Jing W., Fenyong S. (2021). SNHG9 promotes Hepatoblastoma Tumorigenesis via miR-23a-5p/Wnt3a Axis. J. Cancer.

[203] Chen T., Chen J., Zhao X., Zhou J., Sheng Q., Zhu L., Lv Z. (2021). betaKlotho, a direct target of miR-206, contributes to the growth of hepatoblastoma through augmenting PI3K/Akt/mTOR signaling. Am. J. Cancer Res..

[204] Cui X., Liu X., Han Q., Zhu J., Li J., Ren Z., Liu L., Luo Y., Wang Z., Zhang D. (2019). DPEP1 is a direct target of miR-193a-5p and promotes hepatoblastoma progression by PI3K/Akt/mTOR pathway. Cell Death Dis..

[205] Ecevit C.O., Aktas S., Tosun Yildirim H., Demirag B., Erbay A., Karaca I., Celik A., Demir A.B., Ercetin A.P., Olgun N. (2019). MicroRNA-17, MicroRNA-19b, MicroRNA-146a, MicroRNA-302d Expressions in Hepatoblastoma and Clinical Importance. J. Pediatr. Hematol. Oncol..

[206] Gilyazova I., Asadullina D., Kagirova E., Sikka R., Mustafin A., Ivanova E., Bakhtiyarova K., Gilyazova G., Gupta S., Khusnutdinova E. (2023). MiRNA-146a-A Key Player in Immunity and Diseases. Int. J. Mol. Sci..

[207] Liu H.T., Luo C.P., Jiang M.J., Deng Z.J., Teng Y.X., Su J.Y., Pan L.X., Ma L., Guo P.P., Zhong J.H. (2023). miR-17-5p slows progression of hepatocellular carcinoma by downregulating TGFbetaR2. Clin. Transl. Oncol..

[208] von Frowein J., Pagel P., Kappler R., von Schweinitz D., Roscher A., Schmid I. (2011). MicroRNA-492 is processed from the keratin 19 gene and up-regulated in metastatic hepatoblastoma. Hepatology.

[209] Zhang W., Zhu J., Bian J., Zhou C., Zhang S., He S., Lu H., Wang Y., He J. (2025). Association of the *pri-miR-34b/c* rs4938723 T > C polymorphism with hepatoblastoma susceptibility in Eastern Chinese children: A five-center case-control study. BMC Med. Genom..

[210] Wu Z., Chen S., Zuo T., Fu J., Gong J., Liu D., Wang B. (2024). Linc01124 promotes hepatoblastoma proliferation through the miR-24-3p/PI3K/AKT pathway. Gene Rep..

[211] Yuan M.-X., Ji C.-Y., Gao H.-Q., Sheng X.-Y., Xie W.-X., Yin Q. (2021). lncRNA TUG1 regulates angiogenesis via the miR-204-5p/JAK2/STAT3 axis in hepatoblastoma. Mol. Med. Rep..

[212] Zhang W., Liang F., Li Q., Sun H., Li F., Jiao Z., Lei J. (2022). LncRNA MIR205HG accelerates cell proliferation, migration and invasion in hepatoblastoma through the activation of MAPK signaling pathway and PI3K/AKT signaling pathway. Biol. Direct.

[213] Zhu C., He X., Chen K., Huang Z., Yao A., Tian X., You Y., Zeng M. (2021). LncRNA NBR2 aggravates hepatoblastoma cell malignancy and promotes cell proliferation under glucose starvation through the miR-22/TCF7 axis. Cell Cycle.

[214] Jiang W., Ou Z.-L., Zhu Q., Yao Y.-B., Zai H.-Y. (2022). LncRNA OIP5-AS1 aggravates the stemness of hepatoblastoma through recruiting PTBP1 to increase the stability of β-catenin. Pathol.-Res. Pract..

[215] Dong R., Liu X.-Q., Zhang B.-B., Liu B.-H., Zheng S., Dong K.-R. (2017). Long non-coding RNA-CRNDE: A novel regulator of tumor growth and angiogenesis in hepatoblastoma. Oncotarget.

[216] Tan T., Li J., Wen Y., Zou Y., Yang J., Pan J., Hu C., Yao Y., Zhang J., Xin Y. (2021). Association between lncRNA-H19 polymorphisms and hepatoblastoma risk in an ethic Chinese population. J. Cell. Mol. Med..

[217] Galardi A., Colletti M., Palma A., Di Giannatale A. (2022). An update on circular RNA in pediatric cancers. Biomedicines.

[218] Yu J., Yang L., Lu H. (2021). The emerging role of circular RNAs in common solid malignant tumors in children. Cancer Cell Int..

[219] Song H., Bian Z.-X., Li H.-Y., Zhang Y., Ma J., Chen S.-H., Zhu J.-B., Zhang X., Wang J., Gu S. (2019). Characterization of hsa_circ_0000594 as a new biomarker and therapeutic target for hepatoblastoma. Eur. Rev. Med. Pharmacol. Sci..

[220] Zhen N., Gu S., Ma J., Zhu J., Yin M., Xu M., Wang J., Huang N., Cui Z., Bian Z. (2019). CircHMGCS1 promotes hepatoblastoma cell proliferation by regulating the IGF signaling pathway and glutaminolysis. Theranostics.

[221] Zhu Q., Hu Y., Jiang W., Ou Z.-L., Yao Y.-B., Zai H.-Y. (2024). Circ-CCT2 activates wnt/β-catenin signaling to facilitate hepatoblastoma development by stabilizing PTBP1 mRNA. Cell. Mol. Gastroenterol. Hepatol..

[222] Chen F., He L., Qiu L., Zhou Y., Li Z., Chen G., Xin F., Dong X., Xu H., Wang G. (2021). Circular RNA CircEPB41L2 functions as tumor suppressor in hepatocellular carcinoma through sponging miR-590-5p. Cancer Manag. Res..

[223] Han D., Li J., Wang H., Su X., Hou J., Gu Y., Qian C., Lin Y., Liu X., Huang M. (2017). Circular RNA circMTO1 acts as the sponge of microRNA-9 to suppress hepatocellular carcinoma progression. Hepatology.

[224] Liu B.-H., Zhang B.-B., Liu X.-Q., Zheng S., Dong K.-R., Dong R. (2018). Expression profiling identifies circular RNA signature in hepatoblastoma. Cell. Physiol. Biochem..

[225] Xu J., Ji L., Liang Y., Wan Z., Zheng W., Song X., Gorshkov K., Sun Q., Lin H., Zheng X. (2020). CircRNA-SORE mediates sorafenib resistance in hepatocellular carcinoma by stabilizing YBX1. Signal Transduct. Target. Ther..

[226] Mirabello L., Troisi R.J., Savage S.A. (2009). Osteosarcoma incidence and survival rates from 1973 to 2004: Data from the Surveillance, Epidemiology, and End Results Program. Cancer Interdiscip. Int. J. Am. Cancer Soc..

[227] Menendez N., Epelman M., Shao L., Douglas D., Meyers A.B. (2022). Pediatric osteosarcoma: Pearls and pitfalls. Semin. Ultrasound CT MRI.

[228] Meltzer P.S., Helman L.J. (2021). New horizons in the treatment of osteosarcoma. N. Engl. J. Med..

[229] Taran S.J., Taran R., Malipatil N.B. (2017). Pediatric osteosarcoma: An updated review. Indian J. Med. Paediatr. Oncol..

[230] Sharma A., Pettee D., Mella C., Hord C., Brockwell M., Hardy S., Ball H.C., Safadi F.F., Kuerbitz S.J. (2025). Epigenetic Inactivation of RIPK3-Dependent Necroptosis Augments Cisplatin Chemoresistance in Human Osteosarcoma. Int. J. Mol. Sci..

[231] Tippett V.L., Tattersall L., Ab Latif N.B., Shah K.M., Lawson M.A., Gartland A. (2023). The strategy and clinical relevance of in vitro models of MAP resistance in osteosarcoma: A systematic review. Oncogene.

[232] Link M.P., Goorin A.M., Miser A.W., Green A.A., Pratt C.B., Belasco J.B., Pritchard J., Malpas J.S., Baker A.R., Kirkpatrick J.A. (1986). The effect of adjuvant chemotherapy on relapse-free survival in patients with osteosarcoma of the extremity. N. Engl. J. Med..

[233] Smrke A., Anderson P.M., Gulia A., Gennatas S., Huang P.H., Jones R.L. (2021). Future directions in the treatment of osteosarcoma. Cells.

[234] Beird H.C., Bielack S.S., Flanagan A.M., Gill J., Heymann D., Janeway K.A., Livingston J.A., Roberts R.D., Strauss S.J., Gorlick R. (2022). Osteosarcoma. Nat. Rev. Dis. Primers.

[235] De Noon S., Ijaz J., Coorens T.H., Amary F., Ye H., Strobl A., Lyskjær I., Flanagan A.M., Behjati S. (2021). MYC amplifications are common events in childhood osteosarcoma. J. Pathol. Clin. Res..

[236] Mao J., Li H.-M., Huang Z. (2024). Comprehensive analysis of the expression and prognosis for cyclin-dependent protein kinase family in osteosarcoma. Nucleosides Nucleotides Nucleic Acids.

[237] Synoradzki K.J., Bartnik E., Czarnecka A.M., Fiedorowicz M., Firlej W., Brodziak A., Stasinska A., Rutkowski P., Grieb P. (2021). TP53 in biology and treatment of osteosarcoma. Cancers.

[238] Xie L., Yang Y., Guo W., Che D., Xu J., Sun X., Liu K., Ren T., Liu X., Yang Y. (2021). The clinical implications of tumor mutational burden in osteosarcoma. Front. Oncol..

[239] Hu X., Yu A.-X., Qi B.-W., Fu T., Wu G., Zhou M., Luo J., Xu J.-H. (2010). The expression and significance of IDH1 and p53 in osteosarcoma. J. Exp. Clin. Cancer Res..

[240] Doghish A.S., Hegazy M., Ismail A., El-Mahdy H.A., Elsakka E.G., Elkhawaga S.Y., Elkady M.A., Yehia A.M., Abdelmaksoud N.M., Mokhtar M.M. (2023). A spotlight on the interplay of signaling pathways and the role of miRNAs in osteosarcoma pathogenesis and therapeutic resistance. Pathol.-Res. Pract..

[241] Trivedi J., Desai A., Saha P., Ajgaonkar S., Nabar S., Momin M., Muzumdar I., Nair S. (2024). Current insights into signature MicroRNA networks and signal transduction in osteosarcoma. Curr. Pharmacol. Rep..

[242] Liao X., Wei R., Zhou J., Wu K., Li J. (2024). Emerging roles of long non-coding RNAs in osteosarcoma. Front. Mol. Biosci..

[243] Zhao D., Wang S., Chu X., Han D. (2019). LncRNA HIF2PUT inhibited osteosarcoma stem cells proliferation, migration and invasion by regulating HIF2 expression. Artif. Cells Nanomed. Biotechnol..

[244] Li W., He X., Xue R., Zhang Y., Zhang X., Lu J., Zhang Z., Xue L. (2016). Combined over-expression of the hypoxia-inducible factor 2α gene and its long non-coding RNA predicts unfavorable prognosis of patients with osteosarcoma. Pathol.-Res. Pract..

[245] Farzaneh M., Najafi S., Anbiyaee O., Azizidoost S., Khoshnam S.E. (2023). LncRNA MALAT1-related signaling pathways in osteosarcoma. Clin. Transl. Oncol..

[246] Misawa A., Orimo H. (2018). lncRNA HOTAIR inhibits mineralization in osteoblastic osteosarcoma cells by epigenetically repressing ALPL. Calcif. Tissue Int..

[247] Wang B., Qu X.L., Liu J., Lu J., Zhou Z.Y. (2019). HOTAIR promotes osteosarcoma development by sponging miR-217 and targeting ZEB1. J. Cell. Physiol..

[248] Jiang N., Wang X., Xie X., Liao Y., Liu N., Liu J., Miao N., Shen J., Peng T. (2017). lncRNA DANCR promotes tumor progression and cancer stemness features in osteosarcoma by upregulating AXL via miR-33a-5p inhibition. Cancer Lett..

[249] Zhang W., Li J.-Z., Tai Q.-Y., Tang J.-J., Huang Y.-H., Gao S.-B. (2020). LncRNA DANCR regulates osteosarcoma migration and invasion by targeting miR-149/MSI2 axis. Eur. Rev. Med. Pharmacol. Sci..

[250] Ding Q., Mo F., Cai X., Zhang W., Wang J., Yang S., Liu X. (2020). LncRNA CRNDE is activated by SP1 and promotes osteosarcoma proliferation, invasion, and epithelial-mesenchymal transition via Wnt/β-catenin signaling pathway. J. Cell. Biochem..

[251] Ding X., Zhang Y., Liang J., Yin J., Akbar N., Miguel V., Zhou Y. (2022). The long non-coding RNA CRNDE promotes osteosarcoma proliferation and migration by sponging miR-136-5p/MRP9 axis. Ann. Transl. Med..

[252] Li Z., Tang Y., Xing W., Dong W., Wang Z. (2018). LncRNA, CRNDE promotes osteosarcoma cell proliferation, invasion and migration by regulating Notch1 signaling and epithelial-mesenchymal transition. Exp. Mol. Pathol..

[253] Gao Z., Chen S., Ye W. (2025). Cuproptosis related lncRNA signature as a prognostic and therapeutic biomarker in osteosarcoma immunity. Sci. Rep..

[254] Ruan W., Wang P., Feng S., Xue Y., Li Y. (2016). Long non-coding RNA small nucleolar RNA host gene 12 (SNHG12) promotes cell proliferation and migration by upregulating angiomotin gene expression in human osteosarcoma cells. Tumor Biol..

[255] Zhou B., Li L., Li Y., Sun H., Zeng C. (2018). Long noncoding RNA SNHG12 mediates doxorubicin resistance of osteosarcoma via miR-320a/MCL1 axis. Biomed. Pharmacother..

[256] Guan J., He J., Liao S., Wu Z., Lin X., Liu B., Qin X., Tan J., Huang C., Yuan Z. (2022). LncRNA UCA1 accelerates osteosarcoma progression via miR-145 and Wnt/β-catenin pathway. Am. J. Transl. Res..

[257] Zhang Z., Wu X., Han Q., Huang Z. (2021). Downregulation of long non-coding RNA UCA1 represses tumorigenesis and metastasis of osteosarcoma via miR-513b-5p/E2F5 axis. Anti-Cancer Drugs.

[258] Zhang H.-Q., Li T., Li C., Hu H.-T., Zhu S.-M., Lu J.-Q., Chen X.-J., Huang H.-F., Wu Y.-T. (2022). LncRNA THOR promotes endometrial cancer progression through the AKT and ERK signaling pathways. Med. Oncol..

[259] Natua S., Dhamdhere S.G., Mutnuru S.A., Shukla S. (2022). Interplay within tumor microenvironment orchestrates neoplastic RNA metabolism and transcriptome diversity. Wiley Interdiscip. Rev. RNA.

[260] Zhang H., Zhou Q., Shen W. (2022). Circ-FOXM1 promotes the proliferation, migration and EMT process of osteosarcoma cells through FOXM1-mediated Wnt pathway activation. J. Orthop. Surg. Res..

[261] Tang G., Liu L., Xiao Z., Wen S., Chen L., Yang P. (2021). CircRAB3IP upregulates twist family BHLH transcription factor (TWIST1) to promote osteosarcoma progression by sponging miR-580-3p. Bioengineered.

[262] Wang Z., Deng M., Chen L., Wang W., Liu G., Liu D., Han Z., Zhou Y. (2020). Circular RNA Circ-03955 promotes epithelial-mesenchymal transition in osteosarcoma by regulating miR-3662/metadherin pathway. Front. Oncol..

[263] Wang G., Wei X., Gao S., Chen W., Geng Y., Liu J., Guan H. (2023). Circ_LRP6 facilitates osteosarcoma progression via the miR-122-5p/miR-204-5p/HMGB1 axis. Environ. Toxicol..

[264] Yu Y., Dong G., Li Z., Zheng Y., Shi Z., Wang G. (2022). circ-LRP6 contributes to osteosarcoma progression by regulating the miR-141-3p/HDAC4/HMGB1 axis. Int. J. Oncol..

[265] Jin Z., Ye J., Chen S., Ren Y., Guo W. (2022). CircDOCK1 regulates miR-186/DNMT3A to promote osteosarcoma progression. Biomedicines.

[266] Li S., Liu F., Zheng K., Wang W., Qiu E., Pei Y., Wang S., Zhang J., Zhang X. (2021). CircDOCK1 promotes the tumorigenesis and cisplatin resistance of osteogenic sarcoma via the miR-339-3p/IGF1R axis. Mol. Cancer.

[267] Xu G., Zhang H., Shi Y., Yang F. (2022). Circular RNA circDOCK1 contributes to osteosarcoma progression by acting as a ceRNA for miR-936 to regulate LEF1. J. Bone Oncol..

[268] Li X., Zhao X., Li J., Zhang X. (2023). Circ_001422 aggravates osteosarcoma progression through targeting miR-497-5p/E2F3 axis. J. Biochem. Mol. Toxicol..

[269] Yang B., Li L., Tong G., Zeng Z., Tan J., Su Z., Liu Z., Lin J., Gao W., Chen J. (2021). Circular RNA circ_001422 promotes the progression and metastasis of osteosarcoma via the miR-195-5p/FGF2/PI3K/Akt axis. J. Exp. Clin. Cancer Res..

[270] Wan J., Liu Y., Long F., Tian J., Zhang C. (2021). circPVT1 promotes osteosarcoma glycolysis and metastasis by sponging miR-423-5p to activate Wnt5a/Ror2 signaling. Cancer Sci..

[271] Zhou C., Balmer L., Song M., Wu K., Wang W., Wang H. (2024). CircPVT1 promotes migration and invasion by regulating miR-490-5p/HAVCR2 axis in osteosarcoma cells. J. Cell. Mol. Med..

[272] Eaton B.R., Claude L., Indelicato D.J., Vatner R., Yeh B., Schwarz R., Laack N. (2021). Ewing sarcoma. Pediatr. Blood Cancer.

[273] Sole A., Grossetête S., Heintzé M., Babin L., Zaïdi S., Revy P., Renouf B., De Cian A., Giovannangeli C., Pierre-Eugène C. (2021). Unraveling ewing sarcoma tumorigenesis originating from patient-derived mesenchymal stem cells. Cancer Res..

[274] Jo V.Y. (2020). EWSR1 fusions: Ewing sarcoma and beyond. Cancer Cytopathol..

[275] Sbaraglia M., Righi A., Gambarotti M., Dei Tos A.P. (2020). Ewing sarcoma and Ewing-like tumors. Virchows Arch..

[276] Yasir M., Park J., Chun W. (2023). EWS/FLI1 characterization, activation, repression, target genes and therapeutic opportunities in Ewing sarcoma. Int. J. Mol. Sci..

[277] Zöllner S.K., Amatruda J.F., Bauer S., Collaud S., de Álava E., DuBois S.G., Hardes J., Hartmann W., Kovar H., Metzler M. (2021). Ewing sarcoma—Diagnosis, treatment, clinical challenges and future perspectives. J. Clin. Med..

[278] Abboud A., Masrouha K., Saliba M., Haidar R., Saab R., Khoury N., Tawil A., Saghieh S. (2021). Extraskeletal Ewing sarcoma: Diagnosis, management and prognosis. Oncol. Lett..

[279] Gupta A., Riedel R.F., Shah C., Borinstein S.C., Isakoff M.S., Chugh R., Rosenblum J.M., Murphy E.S., Campbell S.R., Albert C.M. (2023). Consensus recommendations in the management of Ewing sarcoma from the National Ewing Sarcoma Tumor Board. Cancer.

[280] Heesen P., Ranft A., Bhadri V., Brichard B., Collaud S., Cyprova S., Eich H., Ek T., Gelderblom H., Hardes J. (2023). Association between local treatment modalities and event-free survival, overall survival, and local recurrence in patients with localised Ewing Sarcoma. Report from the Ewing 2008 trial. Eur. J. Cancer.

[281] Stachelek G.C., Ligon J.A., Vogel J., Levin A.S., Llosa N.J., Ladle B.H., Meyer C.F., Terezakis S.A., Morris C.D., Ladra M.M. (2021). Predictors of recurrence and patterns of initial failure in localized Ewing sarcoma: A contemporary 20-year experience. Sarcoma.

[282] Palmini G., Brandi M.L. (2021). microRNAs and bone tumours: Role of tiny molecules in the development and progression of chondrosarcoma, of giant cell tumour of bone and of Ewing’s sarcoma. Bone.

[283] Buscaglia L.E.B., Li Y. (2011). Apoptosis and the target genes of microRNA-21. Chin. J. Cancer.

[284] Scuderi S.A., Calabrese G., Paterniti I., Campolo M., Lanza M., Capra A.P., Pantaleo L., Munaò S., Colarossi L., Forte S. (2022). The biological function of MicroRNAs in bone tumors. Int. J. Mol. Sci..

[285] Ye C., Yu X., Liu X., Dai M., Zhang B. (2018). miR-30d inhibits cell biological progression of Ewing’s sarcoma by suppressing the MEK/ERK and PI3K/Akt pathways in vitro. Oncol. Lett..

[286] Kawano M., Tanaka K., Itonaga I., Iwasaki T., Tsumura H. (2018). MicroRNA-181c prevents apoptosis by targeting of FAS receptor in Ewing’s sarcoma cells. Cancer Cell Int..

[287] Satterfield L., Shuck R., Kurenbekova L., Allen-Rhoades W., Edwards D., Huang S., Rajapakshe K., Coarfa C., Donehower L.A., Yustein J.T. (2017). miR-130b directly targets ARHGAP1 to drive activation of a metastatic CDC42-PAK1-AP1 positive feedback loop in Ewing sarcoma. Int. J. Cancer.

[288] Zhou X., Chen J., Xiao Q., Wang T., Yu Y., Li B., Shao G., Li Y., Zhang Z. (2018). MicroRNA-638 inhibits cell growth and tubule formation by suppressing VEGFA expression in human Ewing sarcoma cells. Biosci. Rep..

[289] Hassan M., Shahzadi S., Malik A., Din S.u., Yasir M., Chun W., Kloczkowski A. (2023). Oncomeric profiles of microRNAs as new therapeutic targets for treatment of Ewing’s sarcoma: A composite review. Genes.

[290] Hassan M., Malik A., Shahzadi S., Kloczkowski A. (2025). Unveiling Let-7a’s Therapeutic Role in Ewing Sarcoma Through Molecular Docking and Deformation Energy Analysis. Curr. Issues Mol. Biol..

[291] Hameiri-Grossman M., Porat-Klein A., Yaniv I., Ash S., Cohen I.J., Kodman Y., Haklai R., Elad-Sfadia G., Kloog Y., Chepurko E. (2015). The association between let-7, RAS and HIF-1α in Ewing Sarcoma tumor growth. Oncotarget.

[292] McKinsey E., Parrish J., Irwin A., Niemeyer B., Kern H., Birks D., Jedlicka P. (2011). A novel oncogenic mechanism in Ewing sarcoma involving IGF pathway targeting by EWS/Fli1-regulated microRNAs. Oncogene.

[293] Roberto G.M., Vieira G.M., Delsin L.E.A., de Oliveira Silva M., Hakime R.G., Engel E.E., Scrideli C.A., Tone L.G., Brassesco M.S. (2019). MiR-708-5p is inversely associated with EWS/FLI1 Ewing sarcoma but does not represent a prognostic predictor. Cancer Genet..

[294] Chen Z., Wang X., Wang G., Xiao B., Ma Z., Huo H., Li W. (2021). A seven-lncRNA signature for predicting Ewing’s sarcoma. PeerJ.

[295] Palombo R., Frisone P., Fidaleo M., Mercatelli N., Sette C., Paronetto M.P. (2019). The promoter-associated noncoding RNA PNCCCND1_B assembles a protein–RNA complex to regulate cyclin D1 transcription in Ewing sarcoma. Cancer Res..

[296] Xiong J., Wu L., Huang L., Wu C., Liu Z., Deng W., Ma S., Zhou Z., Yu H., Cao K. (2021). LncRNA FOXP4-AS1 promotes progression of ewing sarcoma and is associated with immune infiltrates. Front. Oncol..

[297] Hu L., Huang F., Xu-Qiang L., Hu-Cheng L., Min D., Jin Z. (2021). LncRNA TUG1 promotes Ewing’s sarcoma cell proliferation, migration, and invasion via the miR-199a-3p-MSI2 signaling pathway. Neoplasma.

[298] Siddiqui H., Selich-Taylor J., Felgenhauer J., Otsuru S., Horwitz E., Shah N. (2016). Abstract A48: The lncRNA HOTAIR is overexpressed in Ewing sarcoma and promotes malignant transformation through interactions with histone-modifying complexes. Cancer Res..

[299] He F., Ren T., Tang X. (2023). METTL3-modified lncRNA-MALAT1 regulates the molecular axis of miR-124-3p/CDK4 involved in Ewing’s sarcoma. Cell. Mol. Biol..

[300] Martinelli M., Mancarella C., Scapoli L., Palmieri A., De Sanctis P., Ferrari C., Pasello M., Zucchini C., Scotlandi K. (2022). Polymorphic variants of IGF2BP3 and SENCR have an impact on predisposition and/or progression of Ewing sarcoma. Front. Oncol..

[301] Koppula A., Abdelgawad A., Romero B., Beringer V., Parashar V., Batish M. (2026). Circuitous Ways of EWS::FLI1 Using Circular RNA ZNF609 to Evade Translational Repression by miR-145 in Ewing’s Sarcoma. Biomedicines.

[302] Nawar N., Bukhari S., Adile A.A., Suk Y., Manaswiyoungkul P., Toutah K., Olaoye O.O., Raouf Y.S., Sedighi A., Garcha H.K. (2022). Discovery of HDAC6-selective inhibitor NN-390 with in vitro efficacy in group 3 medulloblastoma. J. Med. Chem..

[303] Good D.J. (2023). Non-coding RNAs in human health and diseases. Genes.

[304] Gouda M.A., Subbiah V. (2023). Expanding the benefit: Dabrafenib/trametinib as tissue-agnostic therapy for BRAF V600E–positive adult and pediatric solid tumors. Am. Soc. Clin. Oncol. Educ. Book.

[305] Manoharan N., Liu K.X., Mueller S., Haas-Kogan D.A., Bandopadhayay P. (2023). Pediatric low-grade glioma: Targeted therapeutics and clinical trials in the molecular era. Neoplasia.

